# Crystal structure of the RuPhos ligand

**DOI:** 10.1107/S2056989021000542

**Published:** 2021-01-26

**Authors:** Kurtis M. Carsch, William Ho, Kai Hin Lui, Gregory Valtierra, Dilek K. Dogutan, Daniel G. Nocera, Shao-Liang Zheng

**Affiliations:** a Department of Chemistry and Chemical Biology, Harvard University, 12 Oxford Street, Cambridge, MA, 02138, USA

**Keywords:** RuPhos, Buchwald ligand, phosphine, cone angle, crystal structure

## Abstract

Palladium 2-di­cyclo­hexyl­phosphanyl-2′,6′-diisopropoxybiphenyl (Pd–RuPhos) catalysts demonstrate high catalytic activity for Negishi cross-couplings of sterically hindered aryl halides, for Suzuki–Miyaura cross-couplings of tosyl­ated olefins, and for Buchwald–Hartwig amination of sterically hindered amines. The solid-state structure of the free RuPhos ligand, C_30_H_43_O_2_P, is reported herein for the first time. RuPhos crystallizes in a triclinic cell containing two independent mol­ecules of the phosphine without any lattice solvent. Pertinent bond metrics and comparisons to other phosphine ligands are presented. The structure of RuPhos will be of assistance in the use of this ligand in the design of cross-coupling catalysts.

## Abstract 2

The solid-state structure of RuPhos (2-di­cyclo­hexyl­phosphanyl-2′,6′-diisopropoxybiphen­yl) is presented for the first time and discussed in detail. The phosphine cone angle is computed and compared to the cone angles of other phosphine ligands.

## Chemical context   

Cross-coupling reactions have emerged as a facile method for C*sp* ^2^—C*sp* ^2^ and C*sp* ^2^—N bond formations. A variety of ancillary phosphine ligands have been observed to mediate challenging Pd-catalyzed cross-coupling reactions (Christmann & Vilar, 2005 ▸). The Pd^0^ reagent Pd_2_(dba)_3_ (dba = di­benz­yl­ideneacetone) in the presence of the ligand 2-di­­cyclo­hexyl­phosphanyl-2′,6′-diisopropoxybiphenyl (RuPhos, see scheme) is especially effective at catalyzing C*sp* ^2^—C*sp* ^2^ bond formation between sterically hindered aryl rings that were previously challenging to couple by traditional cross-coupling methods employing other supporting phosphine ligands (Milne & Buchwald, 2004 ▸). Pd–RuPhos has shown efficacy for a variety of organic substrate transformations, including cross-coupling reactions with sterically hindered aryl halides (Otani *et al.*, 2011 ▸; Carsch *et al.*, 2019 ▸), stereoselective C*sp* ^2^—C*sp* ^2^ bond formation from tosyl­ated olefins (Li *et al.*, 2017 ▸), C*sp* ^2^—N bond formation afforded by the Buchwald–Hartwig amination (Charles *et al.*, 2005 ▸), and in the synthesis of new materials, such as the catalyst-transfer polycondensation to furnish polymeric semiconductors such as poly(3-alkyl­thio­phenes) (Lee *et al.*, 2020 ▸).
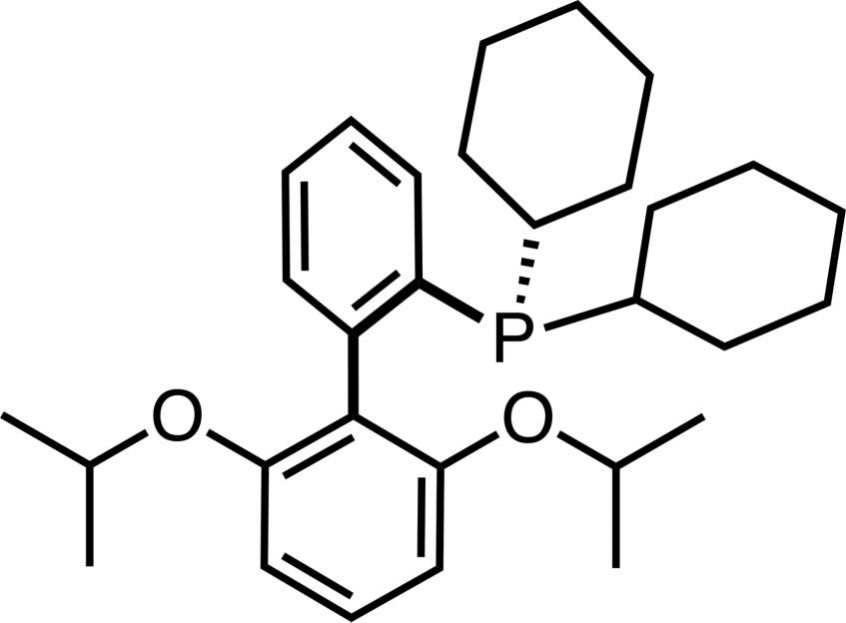


The steric and electronic properties of the ancillary phosphine ligand can have a profound impact on the outcome of the cross-coupling reaction. For example, in the Buchwald–Hartwig amination, Pd–RuPhos displays high catalytic activity for cross-coupling reactions with sterically hindered substrates such as cyclic secondary amines, whereas the related congener, Pd–BrettPhos, demonstrates high catalytic activity with primary amines (Tian *et al.*, 2020 ▸; Charles *et al.*, 2005 ▸). The electronic properties and steric profile of the ligand scaffold impact the elementary steps and catalytic performance of the resulting metal complex (van Leeuwen *et al.*, 2000 ▸). Recent density functional calculations corroborate the importance of ligand properties on the kinetics of cross-coupling chemistry: the rate-limiting step for Pd–RuPhos is predicted to be reductive elimination, while that of the congener Pd–BrettPhos is predicted to be oxidative addition (Tian *et al.*, 2020 ▸). Curiously, the solid-state structure of RuPhos remains absent from the literature. Knowledge of the structural metrics of RuPhos will benefit mechanistic and computational studies of this important ligand and will aid in the rational design of new RuPhos-derivative catalysts.

## Structural commentary   

**Figure 1:**
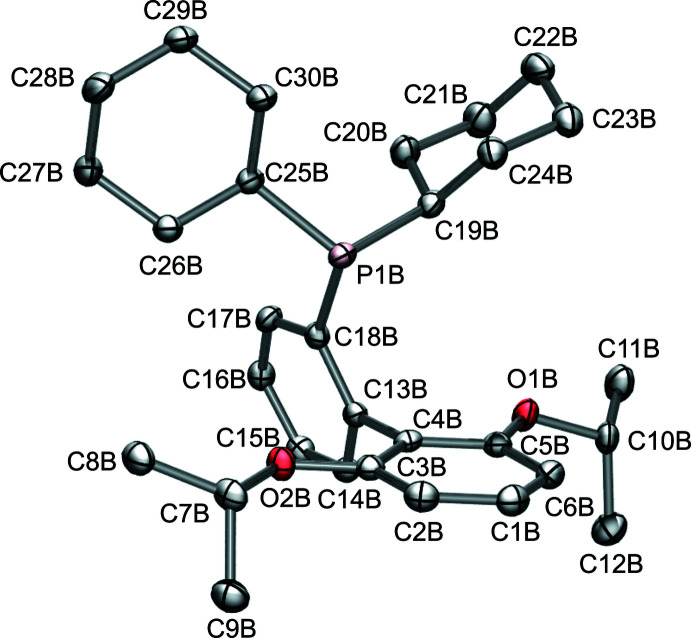
Ellipsoid plot (50% probability ellipsoids) of RuPhos. Hydrogen atoms are omitted for clarity.

The free RuPhos ligand (Fig. 1 ▸) was characterized by single-crystal X-ray diffraction, with pertinent bond metrics listed in Table 1 ▸ and experimental structural details delineated in Table 2 ▸. The asymmetric unit contains two independent mol­ecules, RuPhos A and RuPhos B, which differ modestly in conformation. For conciseness, only the structural metrics of RuPhos B are described hereafter, and RuPhos B is simply referred to as RuPhos. Details of the structural metrics of both mol­ecules in the asymmetric unit can be found in the supporting information.

The C—C bond lengths (Table S3) in the arene rings differ minimally, ranging from 1.385 (2) to 1.402 (2) Å. The P—C*sp* ^2^ and P—C*sp* ^3^ bond lengths (Table 1 ▸) were observed to vary minimally between RuPhos A and RuPhos B. The P—C_Ar_ bond length (P1*B*—C18*B*) is 1.848 (2) Å and it is comparable to the previously reported P-–C_Ar_ bond lengths in PPh_3_ (Samouei *et al.*, 2014 ▸). As expected, the P—C_Cy_ bond lengths are somewhat longer [P1*B*—C19*B*: 1.877 (2) Å; P1*B*—C25*B*: 1.862 (2) Å] and comparable to those observed in PCy_3_ (Davies *et al.*, 1991 ▸). The Cy(C25*B*)—P1*B*—Cy(19B) angle is 105.46 (8)°. The two C_Ar_—P—C_Cy_ angles are 97.03 (8)° (C18*B*—P1*B*—C19*B*) and 101.86 (8)° (C18*B*—P1*B*—C25*B*). The cyclo­hexyl rings each adopt a chair conformation relative to P1*B* and are in an asymmetric orientation relative to the biaryl substituent. No notable inter­actions between the cyclo­hexyl rings and other atoms within RuPhos are observed. Additional electron density close to the phospho­rus is resolved and assigned to a lone pair rather than a light atom based on its proximity to the phospho­rous atom.

The Tolman cone angle qu­anti­fies steric and electronic effects of phosphine ligands (Tolman, 1977 ▸) and is defined as the angle from a hypothetical metal *M* located 2.28 Å from the phospho­rus atom to the van der Waals radii of the outermost atoms of the phosphine ligand. Half angles are defined by the angle between the *M*—P bond and the line between *M*—H_*i*_, where H_*i*_ is the outermost atom on the substituent, calculated as:

θ_*i*_ = *a_i_* + sin ^−1^(*r*_H_/*d_i_*)

where θ_*i*_ is the angle defined between *M*—H_*i*_ and *M*—P and *d_i_* is the distance between *M* and H_*i*_ (Müller & Mingos, 1995 ▸). For unligated RuPhos, the computed Tolman cone angle is 201.53° (Table S5). For comparison, the cone angle for Pd–RuPhos is 198.06° (Arrechea & Buchwald, 2016 ▸). The RuPhos cone angle is larger than those found in PCy_3_ (170°) and PPh_3_ (145°) (Jover & Cirera, 2019 ▸) and is attributed to the steric profile of the biaryl substituent. The cone angle of free RuPhos is larger than the cone angle of Pd–RuPhos, consistent with slight modification of the P hybridization accompanying complexation to the Pd center.

## Supra­molecular features   

**Figure 2:**
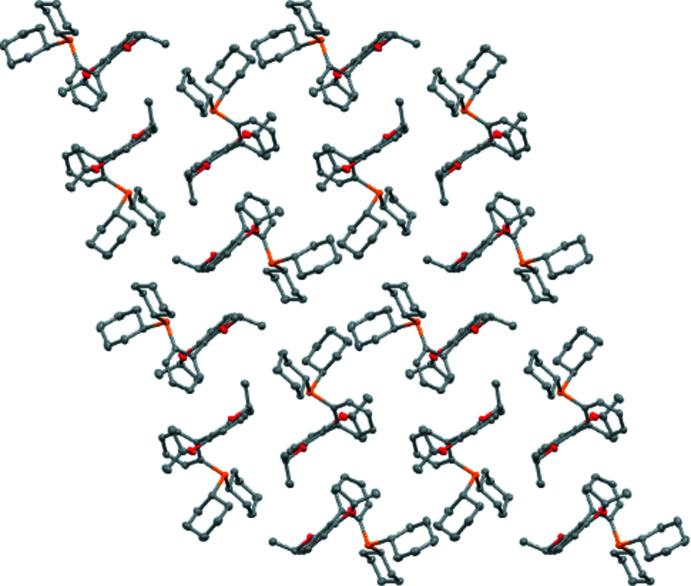
Crystal structure of RuPhos assigned to a parallelepiped geometry, viewed down the *a* axis (*Mercury*; Macrae *et al.*, 2020 ▸). Color scheme: P (orange), C (gray), O (red).

The crystal packing of RuPhos follows a parallelepiped geometry (Fig. 2 ▸), showing two types of inter­molecular channel-like inter­faces, which alternate in parallel planes. In the first type of inter­face channel, cyclo­hexyl substituents from different RuPhos mol­ecules face towards each other. The distance between cyclo­hexyl rings (Table S6) in different unit cells is less than 4 Å [*d*(C20*A*—C22*B*) = 3.942 (3) Å, *d*(C20*A*—C21*B*) = 3.977 (3) Å], consistent with there being no void in the crystal packing. In the second type of channel, biaryl substituents from different RuPhos mol­ecules arrange themselves in a zigzag offset chain pattern (Fig. S2).

Within the asymmetric unit, RuPhos A and RuPhos B are spaced apart by *ca* 3 Å, as defined by the distance between the isopropyl units [H9*BA*⋯H9*AC*: 2.91839 (9) Å]. No void space is observed in the asymmetric unit as evident by a space-filling model (Fig. S3).

The crystal structure of RuPhos shows consistency in atomic composition and connectivity with the reported structure. Coordination by the phosphine to a metal should occlude equatorial ligands on one side of the metal, though less so than its BrettPhos congener would. The small hindrance of Pd–RuPhos is thought to contribute to its high catalytic activity for hindered secondary amines while the larger hindrance of BrettPhos contributes to its high catalytic activity for primary amines (Arrechea & Buchwald, 2016 ▸; Tian *et al.*, 2020 ▸).

The cone angles of free RuPhos and Pd–RuPhos (Arrechea & Buchwald, 2016 ▸) measure 201.54 and 198.07°, respectively. They are smaller than that of free BrettPhos and Pd–BrettPhos (Dikundwar *et al.*, 2017 ▸; DeAngelis *et al.*, 2015 ▸), which are 220.29 and 204.22°, respectively. Because the proportion of *s* character in the lone pair of a phosphine ligand is inversely proportional to the cone angle of the ligand (Tolman, 1977 ▸), the smaller Tolman cone angle of RuPhos implies that RuPhos donates less electron density to its coordinated metal than BrettPhos does. This electronic implication of the RuPhos cone angle corroborates calculations that reductive elimination is the rate-limiting step for Pd–RuPhos-catalyzed couplings (Tian *et al.*, 2020 ▸).

## Database survey   

The structure of the unligated RuPhos ligand has not been previously published according to a search of the Cambridge Structural Database using *ConQuest* 2020.3.0 (CSD, version 5.42, November 2020; Groom *et al.*, 2016 ▸). The structure of metallated Pd^II^ RuPhos has been reported (Arrechea & Buchwald, 2016 ▸).

## Synthesis and crystallization   

RuPhos was purchased from Oakwood Chemical and purified by column chromatography (silica, ethyl acetate). Fractions containing RuPhos were concentrated *in vacuo* and allowed to stand at room temperature under air with slow evaporation for two weeks in a hexa­nes/ethyl acetate (10:1) mixture. Colorless plates were observed (Fig. S1) and employed for data collection.

No evidence for phosphine oxidation was observed in the final refinement. This is attributed to hindered phosphine rotation and the steric profile of the biaryl substituent (Barder *et al.*, 2007 ▸).

## Refinement   

Crystal data, data collection and structure refinement details are summarized in Table 2 ▸. H atoms were placed in calculated positions (C—H = 0.95–1.00 Å) and refined as riding with *U*_iso_(H) = 1.2*U*_eq_(C) or 1.5*U*_eq_(C-meth­yl).

## Supplementary Material

Crystal structure: contains datablock(s) I. DOI: 10.1107/S2056989021000542/mw2173sup1.cif

Structure factors: contains datablock(s) I. DOI: 10.1107/S2056989021000542/mw2173Isup3.hkl

Supporting Information RLVs, bond metrics, structural information. DOI: 10.1107/S2056989021000542/mw2173sup4.pdf

Supporting information file. DOI: 10.1107/S2056989021000542/mw2173Isup4.cml

CCDC reference: 2056274

Additional supporting information:  crystallographic information; 3D view; checkCIF report

## Figures and Tables

**Table 1:** Selected geometric parameters (Å, °) for the two independent mol­ecules RuPhos A and RuPhos B

Bond distances		
C—C Biar­yl	C4—C13	1.495 (2), 1.499 (2)
Ar—P	C18—P1	1.848 (2), 1.848 (2)
Cy—P	C19—P1	1.876 (2), 1.877 (2)
Cy—P	C25—P1	1.865 (2), 1.862 (2)
		
Selected bond angles		
Ar—P—Cy	C18—P1—C25	101.31 (8), 101.86 (8)
Cy—P—Cy	C25—P1—C19	106.07 (8), 105.46 (8)
Ar—P—Cy	C18—P1—C19	98.31 (8), 97.03 (8)
		
Selected torsional angles		
Biar­yl	C3—C4—C13—C14	82.6 (2), 73.2 (2)
Biar­yl	C3—C4—C13—C18	97.6 (2), 105.8 (2)
Biar­yl	C5—C4—C13—C14	96.1 (2), 103.8 (2)
Biar­yl	C5—C4—C13—C18	83.7 (2), 77.2 (2)

**Table 2:** Experimental details

Crystal data	
Chemical formula	C_30_H_43_O_2_P
*M*_r_	466.61
Crystal system, space group	Triclinic, *P* 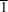
Temperature (K)	100
*a*, *b*, *c* (Å)	9.6160 (4), 15.8209 (7), 19.0324 (9)
α, β, γ (°)	71.2052 (8), 85.1144 (8), 87.9801 (9)
*V* (Å^3^)	2731.0 (2)
*Z*	4
Radiation type	Mo *K*α
μ (mm^−1^)	0.12
Crystal size (mm)	0.42 × 0.24 × 0.12

Data collection	
Diffractometer	Bruker APEXII CCD
Absorption correction	Multi-scan (*SADABS2016/2*; Krause *et al.*, 2015 ▸)
*T*_min_, *T*_max_	0.687, 0.745
No. of measured, independent and observed [*I* > 2σ(*I*)] reflections	55802, 9733, 7694
*R*_int_	0.044
(sin θ/λ)_max_ (Å^−1^)	0.597

Refinement	
*R*[*F* ^2^ > 2σ(*F* ^2^)], *wR*(*F* ^2^), *S*	0.042, 0.116, 1.05
No. of reflections	9733
No. of parameters	603
H-atom treatment	H-atom parameters constrained
Δρ_max_, Δρ_min_ (e Å^−3^)	0.49, −0.27

Computer programs: *APEX3* and *SAINT* (Bruker, 2015 ▸), *SHELXT2014* (Sheldrick, 2015*a* ▸), *SHELXL2018/3* (Sheldrick, 2015*b* ▸), *SHELXTL* (Sheldrick, 2008 ▸), and *Mercury* (Macrae *et al.*, 2020 ▸).

## supplementary crystallographic information

### Crystal data

**Table:**

C_30_H_43_O_2_P	*Z* = 4
*M**_r_* = 466.61	*F*(000) = 1016
Triclinic, *P*1	*D*_x_ = 1.135 Mg m^−^^3^
*a* = 9.6160 (4) Å	Mo *K*α radiation, λ = 0.71073 Å
*b* = 15.8209 (7) Å	Cell parameters from 9987 reflections
*c* = 19.0324 (9) Å	θ = 2.2–24.8°
α = 71.2052 (8)°	µ = 0.12 mm^−^^1^
β = 85.1144 (8)°	*T* = 100 K
γ = 87.9801 (9)°	Plate, colorless
*V* = 2731.0 (2) Å^3^	0.42 × 0.24 × 0.12 mm

### Data collection

**Table:**

Bruker APEXII CCD diffractometer	7694 reflections with *I* > 2σ(*I*)
Radiation source: fine-focus sealed tube	*R*_int_ = 0.044
ω and phi scans	θ_max_ = 25.1°, θ_min_ = 1.4°
Absorption correction: multi-scan (*SADABS2016/2*; Krause *et al.*, 2015)	*h* = −11→11
*T*_min_ = 0.687, *T*_max_ = 0.745	*k* = −18→18
55802 measured reflections	*l* = −22→22
9733 independent reflections	

### Refinement

**Table:**

Refinement on *F*^2^	Primary atom site location: dual
Least-squares matrix: full	Secondary atom site location: difference Fourier map
*R*[*F*^2^ > 2σ(*F*^2^)] = 0.042	Hydrogen site location: inferred from neighbouring sites
*wR*(*F*^2^) = 0.116	H-atom parameters constrained
*S* = 1.05	*w* = 1/[σ^2^(*F*_o_^2^) + (0.0678*P*)^2^ + 0.4429*P*] where *P* = (*F*_o_^2^ + 2*F*_c_^2^)/3
9733 reflections	(Δ/σ)_max_ = 0.001
603 parameters	Δρ_max_ = 0.49 e Å^−^^3^
0 restraints	Δρ_min_ = −0.27 e Å^−^^3^

### Special details

**Table:**

Geometry. All esds (except the esd in the dihedral angle between two l.s. planes) are estimated using the full covariance matrix. The cell esds are taken into account individually in the estimation of esds in distances, angles and torsion angles; correlations between esds in cell parameters are only used when they are defined by crystal symmetry. An approximate (isotropic) treatment of cell esds is used for estimating esds involving l.s. planes.
Refinement. No significant disordering was present.

### Fractional atomic coordinates and isotropic or equivalent isotropic displacement parameters (Å^2^)

**Table:**

	*x*	*y*	*z*	*U*_iso_*/*U*_eq_	
P1A	0.44121 (4)	0.24874 (3)	0.10395 (2)	0.02025 (12)	
O1A	0.73773 (12)	0.26833 (8)	0.22546 (7)	0.0254 (3)	
O2A	0.38076 (12)	0.46762 (8)	0.12987 (7)	0.0260 (3)	
C1A	0.76140 (18)	0.49580 (12)	0.09137 (10)	0.0253 (4)	
H1A	0.828797	0.538226	0.062727	0.030*	
C2A	0.80634 (18)	0.41479 (12)	0.13848 (9)	0.0232 (4)	
H2A	0.903169	0.401717	0.142059	0.028*	
C3A	0.70763 (17)	0.35312 (11)	0.18034 (9)	0.0205 (4)	
C4A	0.56474 (17)	0.37326 (11)	0.17716 (9)	0.0201 (4)	
C5A	0.52256 (17)	0.45552 (11)	0.12887 (9)	0.0212 (4)	
C6A	0.62045 (18)	0.51673 (12)	0.08484 (9)	0.0238 (4)	
H6A	0.591592	0.571762	0.050958	0.029*	
C7A	0.87460 (17)	0.24929 (12)	0.25305 (9)	0.0226 (4)	
H7A	0.947689	0.268124	0.210533	0.027*	
C8A	0.8784 (2)	0.14893 (12)	0.28799 (11)	0.0327 (4)	
H8AA	0.803622	0.130433	0.327973	0.049*	
H8AB	0.968856	0.130520	0.308542	0.049*	
H8AC	0.865337	0.120769	0.250117	0.049*	
C9A	0.8942 (2)	0.29824 (13)	0.30753 (11)	0.0337 (5)	
H9AA	0.881824	0.362507	0.283121	0.051*	
H9AB	0.988436	0.286470	0.324639	0.051*	
H9AC	0.825244	0.277568	0.350252	0.051*	
C10A	0.32140 (18)	0.55063 (11)	0.08509 (10)	0.0234 (4)	
H10A	0.364353	0.567120	0.032781	0.028*	
C11A	0.16772 (18)	0.53064 (12)	0.08720 (10)	0.0286 (4)	
H11A	0.157521	0.479873	0.069211	0.043*	
H11B	0.120351	0.583090	0.055317	0.043*	
H11C	0.125984	0.516023	0.138452	0.043*	
C12A	0.3421 (2)	0.62528 (12)	0.11709 (10)	0.0281 (4)	
H12A	0.302150	0.607829	0.168801	0.042*	
H12B	0.295424	0.679661	0.087671	0.042*	
H12C	0.442088	0.636519	0.115453	0.042*	
C13A	0.45947 (16)	0.30853 (11)	0.22582 (9)	0.0189 (4)	
C14A	0.42997 (18)	0.30783 (12)	0.29927 (9)	0.0244 (4)	
H14A	0.478409	0.347259	0.316983	0.029*	
C15A	0.33181 (18)	0.25092 (12)	0.34640 (9)	0.0243 (4)	
H15A	0.312327	0.251473	0.396033	0.029*	
C16A	0.26173 (17)	0.19284 (12)	0.32077 (9)	0.0228 (4)	
H16A	0.193853	0.153456	0.352835	0.027*	
C17A	0.29094 (17)	0.19236 (11)	0.24816 (9)	0.0219 (4)	
H17A	0.242364	0.152374	0.231117	0.026*	
C18A	0.39021 (16)	0.24941 (11)	0.19962 (9)	0.0191 (4)	
C19A	0.56208 (17)	0.14958 (11)	0.12748 (9)	0.0225 (4)	
H19A	0.623416	0.158877	0.164187	0.027*	
C20A	0.49358 (18)	0.05907 (12)	0.16617 (11)	0.0277 (4)	
H20A	0.430588	0.046087	0.132477	0.033*	
H20B	0.436623	0.061208	0.211426	0.033*	
C21A	0.6032 (2)	−0.01574 (13)	0.18785 (12)	0.0336 (5)	
H21A	0.660653	−0.005816	0.225178	0.040*	
H21B	0.555675	−0.073817	0.210701	0.040*	
C22A	0.6969 (2)	−0.01855 (13)	0.12017 (12)	0.0358 (5)	
H22A	0.768857	−0.065767	0.135702	0.043*	
H22B	0.640685	−0.033274	0.084521	0.043*	
C23A	0.7672 (2)	0.07093 (14)	0.08252 (11)	0.0343 (5)	
H23A	0.824561	0.068569	0.037461	0.041*	
H23B	0.830192	0.082695	0.116769	0.041*	
C24A	0.66063 (19)	0.14738 (14)	0.06047 (10)	0.0317 (4)	
H24A	0.710452	0.204885	0.039698	0.038*	
H24B	0.605455	0.139840	0.021323	0.038*	
C25A	0.27731 (17)	0.20963 (11)	0.07905 (9)	0.0208 (4)	
H25A	0.247972	0.152025	0.117565	0.025*	
C26A	0.16401 (17)	0.28097 (11)	0.07721 (9)	0.0228 (4)	
H26A	0.147661	0.287411	0.127304	0.027*	
H26B	0.197014	0.339067	0.042142	0.027*	
C27A	0.02658 (18)	0.25724 (13)	0.05328 (10)	0.0272 (4)	
H27A	−0.042876	0.305446	0.051484	0.033*	
H27B	−0.010478	0.201445	0.090221	0.033*	
C28A	0.04904 (19)	0.24489 (13)	−0.02343 (10)	0.0287 (4)	
H28A	−0.039501	0.226291	−0.036863	0.034*	
H28B	0.077098	0.302388	−0.061213	0.034*	
C29A	0.16146 (18)	0.17469 (12)	−0.02343 (10)	0.0265 (4)	
H29A	0.178402	0.170659	−0.074302	0.032*	
H29B	0.128080	0.115811	0.010016	0.032*	
C30A	0.29839 (18)	0.19636 (12)	0.00209 (9)	0.0232 (4)	
H30A	0.337947	0.251408	−0.034815	0.028*	
H30B	0.366119	0.147097	0.004464	0.028*	
P1B	1.03926 (4)	0.27391 (3)	0.64698 (2)	0.01980 (12)	
O1B	0.73186 (12)	0.17442 (8)	0.56160 (6)	0.0247 (3)	
O2B	1.08772 (12)	0.37786 (8)	0.45063 (6)	0.0236 (3)	
C1B	0.70695 (18)	0.41179 (12)	0.45028 (9)	0.0243 (4)	
H1B	0.639525	0.457095	0.431652	0.029*	
C2B	0.66229 (18)	0.32608 (12)	0.49049 (9)	0.0232 (4)	
H2B	0.565622	0.312511	0.499134	0.028*	
C3B	0.76187 (17)	0.26046 (11)	0.51789 (9)	0.0198 (4)	
C4B	0.90492 (17)	0.27906 (11)	0.50426 (9)	0.0185 (4)	
C5B	0.94642 (17)	0.36631 (11)	0.46270 (9)	0.0201 (4)	
C6B	0.84761 (18)	0.43300 (12)	0.43657 (9)	0.0238 (4)	
H6B	0.876068	0.492322	0.409687	0.029*	
C7B	0.59642 (17)	0.13792 (12)	0.56147 (10)	0.0239 (4)	
H7B	0.521795	0.179088	0.571536	0.029*	
C8B	0.59304 (19)	0.05138 (13)	0.62519 (11)	0.0335 (5)	
H8BA	0.667611	0.011687	0.615704	0.050*	
H8BB	0.502508	0.022705	0.629810	0.050*	
H8BC	0.606787	0.063489	0.671467	0.050*	
C9B	0.5798 (2)	0.12544 (13)	0.48696 (11)	0.0323 (4)	
H9BA	0.589932	0.183135	0.447410	0.049*	
H9BB	0.487089	0.101298	0.487438	0.049*	
H9BC	0.651476	0.083819	0.477855	0.049*	
C10B	1.14318 (18)	0.46379 (11)	0.40493 (9)	0.0244 (4)	
H10B	1.093593	0.512342	0.420388	0.029*	
C11B	1.29504 (19)	0.46067 (13)	0.42091 (10)	0.0283 (4)	
H11D	1.301510	0.450503	0.474117	0.042*	
H11E	1.339250	0.517506	0.391998	0.042*	
H11F	1.342733	0.411976	0.406959	0.042*	
C12B	1.1272 (2)	0.47910 (13)	0.32287 (10)	0.0301 (4)	
H12D	1.178641	0.432585	0.307474	0.045*	
H12E	1.164566	0.537774	0.293236	0.045*	
H12F	1.028194	0.476797	0.315047	0.045*	
C13B	1.01048 (16)	0.20602 (11)	0.53049 (9)	0.0177 (3)	
C14B	1.03032 (17)	0.14347 (11)	0.49285 (9)	0.0215 (4)	
H14B	0.976273	0.147910	0.452260	0.026*	
C15B	1.12756 (17)	0.07502 (11)	0.51373 (9)	0.0227 (4)	
H15B	1.139771	0.032658	0.487868	0.027*	
C16B	1.20710 (17)	0.06889 (11)	0.57289 (9)	0.0224 (4)	
H16B	1.275321	0.022894	0.587028	0.027*	
C17B	1.18669 (17)	0.13003 (11)	0.61122 (9)	0.0217 (4)	
H17B	1.241577	0.125313	0.651551	0.026*	
C18B	1.08682 (17)	0.19868 (11)	0.59176 (9)	0.0194 (4)	
C19B	0.91541 (17)	0.19627 (11)	0.71832 (9)	0.0223 (4)	
H19B	0.856780	0.169966	0.689800	0.027*	
C20B	0.98146 (18)	0.11693 (12)	0.77526 (10)	0.0286 (4)	
H20C	1.041014	0.082756	0.748730	0.034*	
H20D	1.041788	0.139264	0.804967	0.034*	
C21B	0.8713 (2)	0.05504 (13)	0.82749 (11)	0.0345 (5)	
H21C	0.817380	0.027502	0.798613	0.041*	
H21D	0.918013	0.006570	0.865178	0.041*	
C22B	0.7725 (2)	0.10601 (14)	0.86646 (11)	0.0353 (5)	
H22C	0.699449	0.065258	0.897991	0.042*	
H22D	0.824911	0.128745	0.899127	0.042*	
C23B	0.70455 (19)	0.18366 (13)	0.80992 (11)	0.0320 (4)	
H23C	0.643527	0.217229	0.836379	0.038*	
H23D	0.645504	0.160359	0.780269	0.038*	
C24B	0.81342 (18)	0.24663 (12)	0.75773 (10)	0.0272 (4)	
H24C	0.866091	0.274836	0.786643	0.033*	
H24D	0.765810	0.294559	0.720036	0.033*	
C25B	1.20122 (17)	0.27172 (11)	0.69469 (9)	0.0208 (4)	
H25B	1.228156	0.208342	0.720520	0.025*	
C26B	1.31797 (17)	0.31641 (12)	0.63462 (9)	0.0222 (4)	
H26C	1.334558	0.281686	0.599700	0.027*	
H26D	1.287521	0.377284	0.605919	0.027*	
C27B	1.45393 (18)	0.32211 (13)	0.66890 (10)	0.0282 (4)	
H27C	1.524993	0.353401	0.629089	0.034*	
H27D	1.489293	0.261125	0.693694	0.034*	
C28B	1.4312 (2)	0.37213 (14)	0.72561 (10)	0.0321 (4)	
H28C	1.519260	0.372367	0.748948	0.039*	
H28D	1.404605	0.434862	0.699914	0.039*	
C29B	1.31674 (19)	0.32821 (13)	0.78577 (10)	0.0289 (4)	
H29C	1.300406	0.363504	0.820269	0.035*	
H29D	1.347692	0.267590	0.814789	0.035*	
C30B	1.18020 (18)	0.32175 (12)	0.75184 (9)	0.0245 (4)	
H30C	1.144103	0.382614	0.727317	0.029*	
H30D	1.109851	0.290248	0.791983	0.029*	

### Atomic displacement parameters (Å^2^)

**Table:**

	*U*^11^	*U*^22^	*U*^33^	*U*^12^	*U*^13^	*U*^23^
P1A	0.0213 (2)	0.0221 (2)	0.0174 (2)	−0.00280 (18)	−0.00069 (17)	−0.00635 (18)
O1A	0.0190 (6)	0.0217 (7)	0.0317 (7)	−0.0014 (5)	−0.0075 (5)	−0.0017 (5)
O2A	0.0212 (6)	0.0212 (7)	0.0308 (7)	0.0014 (5)	−0.0038 (5)	−0.0016 (5)
C1A	0.0266 (9)	0.0263 (10)	0.0233 (9)	−0.0079 (8)	0.0009 (7)	−0.0082 (8)
C2A	0.0209 (9)	0.0256 (10)	0.0242 (9)	−0.0019 (7)	−0.0023 (7)	−0.0092 (8)
C3A	0.0228 (9)	0.0212 (9)	0.0187 (8)	−0.0007 (7)	−0.0051 (7)	−0.0070 (7)
C4A	0.0230 (9)	0.0201 (9)	0.0188 (8)	−0.0012 (7)	−0.0026 (7)	−0.0081 (7)
C5A	0.0212 (9)	0.0226 (9)	0.0214 (9)	−0.0003 (7)	−0.0021 (7)	−0.0092 (7)
C6A	0.0287 (10)	0.0217 (9)	0.0206 (9)	−0.0019 (7)	−0.0018 (7)	−0.0058 (7)
C7A	0.0172 (8)	0.0266 (10)	0.0229 (9)	0.0015 (7)	−0.0047 (7)	−0.0059 (8)
C8A	0.0289 (10)	0.0289 (11)	0.0387 (11)	0.0028 (8)	−0.0109 (8)	−0.0068 (9)
C9A	0.0408 (11)	0.0334 (11)	0.0284 (10)	0.0036 (9)	−0.0133 (9)	−0.0099 (9)
C10A	0.0273 (9)	0.0200 (9)	0.0209 (9)	0.0036 (7)	−0.0048 (7)	−0.0035 (7)
C11A	0.0271 (10)	0.0286 (10)	0.0315 (10)	0.0048 (8)	−0.0072 (8)	−0.0109 (8)
C12A	0.0328 (10)	0.0269 (10)	0.0256 (10)	0.0008 (8)	−0.0022 (8)	−0.0099 (8)
C13A	0.0183 (8)	0.0171 (9)	0.0202 (8)	0.0036 (7)	−0.0040 (7)	−0.0043 (7)
C14A	0.0260 (9)	0.0259 (10)	0.0235 (9)	−0.0003 (7)	−0.0045 (7)	−0.0103 (8)
C15A	0.0264 (9)	0.0293 (10)	0.0173 (9)	0.0024 (8)	−0.0030 (7)	−0.0076 (8)
C16A	0.0206 (9)	0.0240 (9)	0.0200 (9)	−0.0010 (7)	0.0011 (7)	−0.0025 (7)
C17A	0.0212 (9)	0.0223 (9)	0.0228 (9)	−0.0019 (7)	−0.0037 (7)	−0.0075 (7)
C18A	0.0192 (8)	0.0204 (9)	0.0171 (8)	0.0029 (7)	−0.0031 (7)	−0.0049 (7)
C19A	0.0211 (9)	0.0258 (10)	0.0217 (9)	0.0004 (7)	−0.0033 (7)	−0.0089 (7)
C20A	0.0243 (9)	0.0233 (10)	0.0349 (10)	−0.0005 (7)	−0.0029 (8)	−0.0085 (8)
C21A	0.0304 (10)	0.0241 (10)	0.0454 (12)	0.0001 (8)	−0.0067 (9)	−0.0091 (9)
C22A	0.0331 (11)	0.0382 (12)	0.0455 (12)	0.0123 (9)	−0.0159 (9)	−0.0246 (10)
C23A	0.0276 (10)	0.0470 (13)	0.0282 (10)	0.0096 (9)	−0.0017 (8)	−0.0130 (9)
C24A	0.0287 (10)	0.0406 (12)	0.0240 (10)	0.0065 (9)	−0.0001 (8)	−0.0092 (9)
C25A	0.0229 (9)	0.0207 (9)	0.0193 (9)	−0.0012 (7)	−0.0018 (7)	−0.0068 (7)
C26A	0.0263 (9)	0.0224 (9)	0.0197 (9)	0.0015 (7)	−0.0021 (7)	−0.0070 (7)
C27A	0.0252 (9)	0.0332 (11)	0.0260 (10)	0.0055 (8)	−0.0063 (7)	−0.0127 (8)
C28A	0.0260 (10)	0.0339 (11)	0.0284 (10)	0.0035 (8)	−0.0081 (8)	−0.0122 (8)
C29A	0.0299 (10)	0.0300 (10)	0.0231 (9)	0.0006 (8)	−0.0053 (7)	−0.0127 (8)
C30A	0.0245 (9)	0.0255 (10)	0.0224 (9)	0.0020 (7)	−0.0047 (7)	−0.0110 (8)
P1B	0.0204 (2)	0.0215 (2)	0.0189 (2)	0.00027 (18)	−0.00450 (17)	−0.00770 (18)
O1B	0.0187 (6)	0.0236 (7)	0.0270 (7)	−0.0037 (5)	−0.0050 (5)	−0.0001 (5)
O2B	0.0223 (6)	0.0189 (6)	0.0257 (6)	−0.0036 (5)	0.0004 (5)	−0.0021 (5)
C1B	0.0262 (9)	0.0231 (10)	0.0243 (9)	0.0051 (7)	−0.0043 (7)	−0.0083 (8)
C2B	0.0205 (9)	0.0282 (10)	0.0216 (9)	0.0009 (7)	−0.0032 (7)	−0.0086 (8)
C3B	0.0234 (9)	0.0197 (9)	0.0166 (8)	−0.0019 (7)	−0.0022 (7)	−0.0057 (7)
C4B	0.0216 (9)	0.0205 (9)	0.0152 (8)	0.0001 (7)	−0.0037 (6)	−0.0078 (7)
C5B	0.0226 (9)	0.0218 (9)	0.0173 (8)	−0.0015 (7)	−0.0019 (7)	−0.0078 (7)
C6B	0.0295 (10)	0.0184 (9)	0.0225 (9)	−0.0001 (7)	−0.0023 (7)	−0.0049 (7)
C7B	0.0153 (8)	0.0259 (10)	0.0280 (9)	−0.0026 (7)	−0.0007 (7)	−0.0049 (8)
C8B	0.0259 (10)	0.0323 (11)	0.0360 (11)	−0.0073 (8)	−0.0033 (8)	−0.0011 (9)
C9B	0.0337 (11)	0.0306 (11)	0.0344 (11)	−0.0029 (8)	−0.0074 (8)	−0.0114 (9)
C10B	0.0308 (10)	0.0171 (9)	0.0234 (9)	−0.0063 (7)	0.0022 (7)	−0.0044 (7)
C11B	0.0302 (10)	0.0286 (10)	0.0269 (10)	−0.0099 (8)	0.0027 (8)	−0.0101 (8)
C12B	0.0358 (11)	0.0266 (10)	0.0250 (10)	−0.0033 (8)	0.0004 (8)	−0.0048 (8)
C13B	0.0183 (8)	0.0167 (9)	0.0167 (8)	−0.0039 (7)	−0.0002 (6)	−0.0032 (7)
C14B	0.0224 (9)	0.0241 (9)	0.0177 (8)	−0.0049 (7)	−0.0016 (7)	−0.0059 (7)
C15B	0.0250 (9)	0.0202 (9)	0.0236 (9)	−0.0025 (7)	0.0007 (7)	−0.0086 (7)
C16B	0.0212 (9)	0.0184 (9)	0.0255 (9)	0.0003 (7)	−0.0018 (7)	−0.0041 (7)
C17B	0.0198 (9)	0.0239 (9)	0.0208 (9)	−0.0013 (7)	−0.0058 (7)	−0.0053 (7)
C18B	0.0186 (8)	0.0195 (9)	0.0199 (8)	−0.0033 (7)	0.0003 (7)	−0.0061 (7)
C19B	0.0202 (9)	0.0247 (9)	0.0242 (9)	−0.0017 (7)	−0.0044 (7)	−0.0098 (8)
C20B	0.0245 (9)	0.0296 (10)	0.0278 (10)	−0.0016 (8)	−0.0026 (8)	−0.0036 (8)
C21B	0.0325 (11)	0.0329 (11)	0.0313 (11)	−0.0061 (9)	−0.0015 (8)	−0.0003 (9)
C22B	0.0346 (11)	0.0451 (13)	0.0270 (10)	−0.0155 (9)	0.0035 (8)	−0.0124 (9)
C23B	0.0267 (10)	0.0392 (12)	0.0353 (11)	−0.0069 (8)	0.0036 (8)	−0.0200 (9)
C24B	0.0231 (9)	0.0311 (10)	0.0300 (10)	−0.0011 (8)	0.0000 (8)	−0.0138 (8)
C25B	0.0212 (9)	0.0224 (9)	0.0194 (9)	−0.0008 (7)	−0.0036 (7)	−0.0069 (7)
C26B	0.0241 (9)	0.0236 (9)	0.0191 (9)	−0.0035 (7)	−0.0017 (7)	−0.0067 (7)
C27B	0.0232 (9)	0.0362 (11)	0.0243 (9)	−0.0073 (8)	−0.0010 (7)	−0.0081 (8)
C28B	0.0306 (10)	0.0409 (12)	0.0262 (10)	−0.0132 (9)	−0.0050 (8)	−0.0105 (9)
C29B	0.0308 (10)	0.0378 (11)	0.0199 (9)	−0.0059 (8)	−0.0041 (8)	−0.0108 (8)
C30B	0.0251 (9)	0.0303 (10)	0.0201 (9)	−0.0047 (8)	−0.0026 (7)	−0.0102 (8)

### Geometric parameters (Å, º)

**Table:**

P1A—C18A	1.8482 (16)	P1B—C18B	1.8482 (17)
P1A—C25A	1.8645 (17)	P1B—C25B	1.8624 (17)
P1A—C19A	1.8762 (17)	P1B—C19B	1.8771 (17)
O1A—C3A	1.376 (2)	O1B—C3B	1.373 (2)
O1A—C7A	1.4444 (19)	O1B—C7B	1.4437 (19)
O2A—C5A	1.370 (2)	O2B—C5B	1.367 (2)
O2A—C10A	1.444 (2)	O2B—C10B	1.449 (2)
C1A—C2A	1.386 (3)	C1B—C2B	1.388 (2)
C1A—C6A	1.390 (2)	C1B—C6B	1.388 (2)
C1A—H1A	0.9500	C1B—H1B	0.9500
C2A—C3A	1.387 (2)	C2B—C3B	1.390 (2)
C2A—H2A	0.9500	C2B—H2B	0.9500
C3A—C4A	1.401 (2)	C3B—C4B	1.402 (2)
C4A—C5A	1.399 (2)	C4B—C5B	1.404 (2)
C4A—C13A	1.495 (2)	C4B—C13B	1.499 (2)
C5A—C6A	1.390 (2)	C5B—C6B	1.391 (2)
C6A—H6A	0.9500	C6B—H6B	0.9500
C7A—C9A	1.507 (2)	C7B—C8B	1.508 (2)
C7A—C8A	1.511 (2)	C7B—C9B	1.516 (3)
C7A—H7A	1.0000	C7B—H7B	1.0000
C8A—H8AA	0.9800	C8B—H8BA	0.9800
C8A—H8AB	0.9800	C8B—H8BB	0.9800
C8A—H8AC	0.9800	C8B—H8BC	0.9800
C9A—H9AA	0.9800	C9B—H9BA	0.9800
C9A—H9AB	0.9800	C9B—H9BB	0.9800
C9A—H9AC	0.9800	C9B—H9BC	0.9800
C10A—C11A	1.517 (2)	C10B—C11B	1.513 (2)
C10A—C12A	1.518 (2)	C10B—C12B	1.522 (2)
C10A—H10A	1.0000	C10B—H10B	1.0000
C11A—H11A	0.9800	C11B—H11D	0.9800
C11A—H11B	0.9800	C11B—H11E	0.9800
C11A—H11C	0.9800	C11B—H11F	0.9800
C12A—H12A	0.9800	C12B—H12D	0.9800
C12A—H12B	0.9800	C12B—H12E	0.9800
C12A—H12C	0.9800	C12B—H12F	0.9800
C13A—C14A	1.399 (2)	C13B—C14B	1.396 (2)
C13A—C18A	1.403 (2)	C13B—C18B	1.402 (2)
C14A—C15A	1.379 (2)	C14B—C15B	1.385 (2)
C14A—H14A	0.9500	C14B—H14B	0.9500
C15A—C16A	1.387 (2)	C15B—C16B	1.390 (2)
C15A—H15A	0.9500	C15B—H15B	0.9500
C16A—C17A	1.389 (2)	C16B—C17B	1.387 (2)
C16A—H16A	0.9500	C16B—H16B	0.9500
C17A—C18A	1.397 (2)	C17B—C18B	1.402 (2)
C17A—H17A	0.9500	C17B—H17B	0.9500
C19A—C20A	1.527 (2)	C19B—C20B	1.529 (2)
C19A—C24A	1.532 (2)	C19B—C24B	1.538 (2)
C19A—H19A	1.0000	C19B—H19B	1.0000
C20A—C21A	1.534 (3)	C20B—C21B	1.528 (2)
C20A—H20A	0.9900	C20B—H20C	0.9900
C20A—H20B	0.9900	C20B—H20D	0.9900
C21A—C22A	1.520 (3)	C21B—C22B	1.521 (3)
C21A—H21A	0.9900	C21B—H21C	0.9900
C21A—H21B	0.9900	C21B—H21D	0.9900
C22A—C23A	1.518 (3)	C22B—C23B	1.518 (3)
C22A—H22A	0.9900	C22B—H22C	0.9900
C22A—H22B	0.9900	C22B—H22D	0.9900
C23A—C24A	1.532 (3)	C23B—C24B	1.528 (2)
C23A—H23A	0.9900	C23B—H23C	0.9900
C23A—H23B	0.9900	C23B—H23D	0.9900
C24A—H24A	0.9900	C24B—H24C	0.9900
C24A—H24B	0.9900	C24B—H24D	0.9900
C25A—C26A	1.535 (2)	C25B—C30B	1.535 (2)
C25A—C30A	1.541 (2)	C25B—C26B	1.544 (2)
C25A—H25A	1.0000	C25B—H25B	1.0000
C26A—C27A	1.531 (2)	C26B—C27B	1.528 (2)
C26A—H26A	0.9900	C26B—H26C	0.9900
C26A—H26B	0.9900	C26B—H26D	0.9900
C27A—C28A	1.531 (2)	C27B—C28B	1.529 (3)
C27A—H27A	0.9900	C27B—H27C	0.9900
C27A—H27B	0.9900	C27B—H27D	0.9900
C28A—C29A	1.522 (2)	C28B—C29B	1.527 (2)
C28A—H28A	0.9900	C28B—H28C	0.9900
C28A—H28B	0.9900	C28B—H28D	0.9900
C29A—C30A	1.528 (2)	C29B—C30B	1.532 (2)
C29A—H29A	0.9900	C29B—H29C	0.9900
C29A—H29B	0.9900	C29B—H29D	0.9900
C30A—H30A	0.9900	C30B—H30C	0.9900
C30A—H30B	0.9900	C30B—H30D	0.9900
			
C18A—P1A—C25A	101.31 (7)	C18B—P1B—C25B	101.86 (8)
C18A—P1A—C19A	98.31 (7)	C18B—P1B—C19B	97.03 (7)
C25A—P1A—C19A	106.07 (8)	C25B—P1B—C19B	105.46 (8)
C3A—O1A—C7A	119.19 (12)	C3B—O1B—C7B	119.71 (13)
C5A—O2A—C10A	120.37 (13)	C5B—O2B—C10B	119.60 (13)
C2A—C1A—C6A	121.85 (16)	C2B—C1B—C6B	121.67 (16)
C2A—C1A—H1A	119.1	C2B—C1B—H1B	119.2
C6A—C1A—H1A	119.1	C6B—C1B—H1B	119.2
C1A—C2A—C3A	118.90 (16)	C1B—C2B—C3B	118.66 (16)
C1A—C2A—H2A	120.6	C1B—C2B—H2B	120.7
C3A—C2A—H2A	120.6	C3B—C2B—H2B	120.7
O1A—C3A—C2A	124.80 (15)	O1B—C3B—C2B	124.58 (15)
O1A—C3A—C4A	114.40 (14)	O1B—C3B—C4B	114.10 (14)
C2A—C3A—C4A	120.76 (15)	C2B—C3B—C4B	121.29 (15)
C5A—C4A—C3A	118.97 (15)	C3B—C4B—C5B	118.52 (15)
C5A—C4A—C13A	120.65 (15)	C3B—C4B—C13B	120.34 (14)
C3A—C4A—C13A	120.36 (15)	C5B—C4B—C13B	121.08 (14)
O2A—C5A—C6A	125.33 (15)	O2B—C5B—C6B	124.82 (15)
O2A—C5A—C4A	113.90 (14)	O2B—C5B—C4B	114.52 (14)
C6A—C5A—C4A	120.76 (16)	C6B—C5B—C4B	120.66 (15)
C1A—C6A—C5A	118.68 (16)	C1B—C6B—C5B	119.18 (16)
C1A—C6A—H6A	120.7	C1B—C6B—H6B	120.4
C5A—C6A—H6A	120.7	C5B—C6B—H6B	120.4
O1A—C7A—C9A	110.12 (14)	O1B—C7B—C8B	104.22 (14)
O1A—C7A—C8A	104.46 (13)	O1B—C7B—C9B	110.32 (14)
C9A—C7A—C8A	113.08 (15)	C8B—C7B—C9B	113.21 (16)
O1A—C7A—H7A	109.7	O1B—C7B—H7B	109.6
C9A—C7A—H7A	109.7	C8B—C7B—H7B	109.6
C8A—C7A—H7A	109.7	C9B—C7B—H7B	109.6
C7A—C8A—H8AA	109.5	C7B—C8B—H8BA	109.5
C7A—C8A—H8AB	109.5	C7B—C8B—H8BB	109.5
H8AA—C8A—H8AB	109.5	H8BA—C8B—H8BB	109.5
C7A—C8A—H8AC	109.5	C7B—C8B—H8BC	109.5
H8AA—C8A—H8AC	109.5	H8BA—C8B—H8BC	109.5
H8AB—C8A—H8AC	109.5	H8BB—C8B—H8BC	109.5
C7A—C9A—H9AA	109.5	C7B—C9B—H9BA	109.5
C7A—C9A—H9AB	109.5	C7B—C9B—H9BB	109.5
H9AA—C9A—H9AB	109.5	H9BA—C9B—H9BB	109.5
C7A—C9A—H9AC	109.5	C7B—C9B—H9BC	109.5
H9AA—C9A—H9AC	109.5	H9BA—C9B—H9BC	109.5
H9AB—C9A—H9AC	109.5	H9BB—C9B—H9BC	109.5
O2A—C10A—C11A	104.29 (13)	O2B—C10B—C11B	105.04 (14)
O2A—C10A—C12A	111.20 (14)	O2B—C10B—C12B	111.04 (14)
C11A—C10A—C12A	111.12 (15)	C11B—C10B—C12B	111.61 (15)
O2A—C10A—H10A	110.0	O2B—C10B—H10B	109.7
C11A—C10A—H10A	110.0	C11B—C10B—H10B	109.7
C12A—C10A—H10A	110.0	C12B—C10B—H10B	109.7
C10A—C11A—H11A	109.5	C10B—C11B—H11D	109.5
C10A—C11A—H11B	109.5	C10B—C11B—H11E	109.5
H11A—C11A—H11B	109.5	H11D—C11B—H11E	109.5
C10A—C11A—H11C	109.5	C10B—C11B—H11F	109.5
H11A—C11A—H11C	109.5	H11D—C11B—H11F	109.5
H11B—C11A—H11C	109.5	H11E—C11B—H11F	109.5
C10A—C12A—H12A	109.5	C10B—C12B—H12D	109.5
C10A—C12A—H12B	109.5	C10B—C12B—H12E	109.5
H12A—C12A—H12B	109.5	H12D—C12B—H12E	109.5
C10A—C12A—H12C	109.5	C10B—C12B—H12F	109.5
H12A—C12A—H12C	109.5	H12D—C12B—H12F	109.5
H12B—C12A—H12C	109.5	H12E—C12B—H12F	109.5
C14A—C13A—C18A	119.78 (15)	C14B—C13B—C18B	119.97 (15)
C14A—C13A—C4A	118.46 (15)	C14B—C13B—C4B	118.23 (14)
C18A—C13A—C4A	121.76 (14)	C18B—C13B—C4B	121.79 (14)
C15A—C14A—C13A	121.16 (16)	C15B—C14B—C13B	121.06 (16)
C15A—C14A—H14A	119.4	C15B—C14B—H14B	119.5
C13A—C14A—H14A	119.4	C13B—C14B—H14B	119.5
C14A—C15A—C16A	119.48 (16)	C14B—C15B—C16B	119.40 (16)
C14A—C15A—H15A	120.3	C14B—C15B—H15B	120.3
C16A—C15A—H15A	120.3	C16B—C15B—H15B	120.3
C15A—C16A—C17A	119.91 (15)	C17B—C16B—C15B	119.89 (16)
C15A—C16A—H16A	120.0	C17B—C16B—H16B	120.1
C17A—C16A—H16A	120.0	C15B—C16B—H16B	120.1
C16A—C17A—C18A	121.45 (16)	C16B—C17B—C18B	121.45 (16)
C16A—C17A—H17A	119.3	C16B—C17B—H17B	119.3
C18A—C17A—H17A	119.3	C18B—C17B—H17B	119.3
C17A—C18A—C13A	118.21 (15)	C13B—C18B—C17B	118.16 (15)
C17A—C18A—P1A	123.97 (13)	C13B—C18B—P1B	117.90 (12)
C13A—C18A—P1A	117.78 (12)	C17B—C18B—P1B	123.72 (13)
C20A—C19A—C24A	111.30 (15)	C20B—C19B—C24B	110.52 (15)
C20A—C19A—P1A	116.03 (12)	C20B—C19B—P1B	116.21 (12)
C24A—C19A—P1A	112.09 (12)	C24B—C19B—P1B	111.95 (12)
C20A—C19A—H19A	105.5	C20B—C19B—H19B	105.8
C24A—C19A—H19A	105.5	C24B—C19B—H19B	105.8
P1A—C19A—H19A	105.5	P1B—C19B—H19B	105.8
C19A—C20A—C21A	111.30 (14)	C21B—C20B—C19B	111.89 (15)
C19A—C20A—H20A	109.4	C21B—C20B—H20C	109.2
C21A—C20A—H20A	109.4	C19B—C20B—H20C	109.2
C19A—C20A—H20B	109.4	C21B—C20B—H20D	109.2
C21A—C20A—H20B	109.4	C19B—C20B—H20D	109.2
H20A—C20A—H20B	108.0	H20C—C20B—H20D	107.9
C22A—C21A—C20A	110.94 (16)	C22B—C21B—C20B	111.02 (16)
C22A—C21A—H21A	109.5	C22B—C21B—H21C	109.4
C20A—C21A—H21A	109.5	C20B—C21B—H21C	109.4
C22A—C21A—H21B	109.5	C22B—C21B—H21D	109.4
C20A—C21A—H21B	109.5	C20B—C21B—H21D	109.4
H21A—C21A—H21B	108.0	H21C—C21B—H21D	108.0
C23A—C22A—C21A	110.46 (16)	C23B—C22B—C21B	110.62 (16)
C23A—C22A—H22A	109.6	C23B—C22B—H22C	109.5
C21A—C22A—H22A	109.6	C21B—C22B—H22C	109.5
C23A—C22A—H22B	109.6	C23B—C22B—H22D	109.5
C21A—C22A—H22B	109.6	C21B—C22B—H22D	109.5
H22A—C22A—H22B	108.1	H22C—C22B—H22D	108.1
C22A—C23A—C24A	111.86 (16)	C22B—C23B—C24B	111.55 (15)
C22A—C23A—H23A	109.2	C22B—C23B—H23C	109.3
C24A—C23A—H23A	109.2	C24B—C23B—H23C	109.3
C22A—C23A—H23B	109.2	C22B—C23B—H23D	109.3
C24A—C23A—H23B	109.2	C24B—C23B—H23D	109.3
H23A—C23A—H23B	107.9	H23C—C23B—H23D	108.0
C19A—C24A—C23A	111.26 (15)	C23B—C24B—C19B	111.15 (15)
C19A—C24A—H24A	109.4	C23B—C24B—H24C	109.4
C23A—C24A—H24A	109.4	C19B—C24B—H24C	109.4
C19A—C24A—H24B	109.4	C23B—C24B—H24D	109.4
C23A—C24A—H24B	109.4	C19B—C24B—H24D	109.4
H24A—C24A—H24B	108.0	H24C—C24B—H24D	108.0
C26A—C25A—C30A	109.39 (14)	C30B—C25B—C26B	109.85 (14)
C26A—C25A—P1A	107.90 (11)	C30B—C25B—P1B	111.27 (11)
C30A—C25A—P1A	110.88 (11)	C26B—C25B—P1B	107.78 (11)
C26A—C25A—H25A	109.5	C30B—C25B—H25B	109.3
C30A—C25A—H25A	109.5	C26B—C25B—H25B	109.3
P1A—C25A—H25A	109.5	P1B—C25B—H25B	109.3
C27A—C26A—C25A	111.96 (14)	C27B—C26B—C25B	111.70 (14)
C27A—C26A—H26A	109.2	C27B—C26B—H26C	109.3
C25A—C26A—H26A	109.2	C25B—C26B—H26C	109.3
C27A—C26A—H26B	109.2	C27B—C26B—H26D	109.3
C25A—C26A—H26B	109.2	C25B—C26B—H26D	109.3
H26A—C26A—H26B	107.9	H26C—C26B—H26D	107.9
C26A—C27A—C28A	110.49 (14)	C26B—C27B—C28B	110.84 (15)
C26A—C27A—H27A	109.6	C26B—C27B—H27C	109.5
C28A—C27A—H27A	109.6	C28B—C27B—H27C	109.5
C26A—C27A—H27B	109.6	C26B—C27B—H27D	109.5
C28A—C27A—H27B	109.6	C28B—C27B—H27D	109.5
H27A—C27A—H27B	108.1	H27C—C27B—H27D	108.1
C29A—C28A—C27A	110.67 (15)	C29B—C28B—C27B	110.81 (15)
C29A—C28A—H28A	109.5	C29B—C28B—H28C	109.5
C27A—C28A—H28A	109.5	C27B—C28B—H28C	109.5
C29A—C28A—H28B	109.5	C29B—C28B—H28D	109.5
C27A—C28A—H28B	109.5	C27B—C28B—H28D	109.5
H28A—C28A—H28B	108.1	H28C—C28B—H28D	108.1
C28A—C29A—C30A	112.09 (15)	C28B—C29B—C30B	111.36 (15)
C28A—C29A—H29A	109.2	C28B—C29B—H29C	109.4
C30A—C29A—H29A	109.2	C30B—C29B—H29C	109.4
C28A—C29A—H29B	109.2	C28B—C29B—H29D	109.4
C30A—C29A—H29B	109.2	C30B—C29B—H29D	109.4
H29A—C29A—H29B	107.9	H29C—C29B—H29D	108.0
C29A—C30A—C25A	111.79 (14)	C29B—C30B—C25B	111.57 (14)
C29A—C30A—H30A	109.3	C29B—C30B—H30C	109.3
C25A—C30A—H30A	109.3	C25B—C30B—H30C	109.3
C29A—C30A—H30B	109.3	C29B—C30B—H30D	109.3
C25A—C30A—H30B	109.3	C25B—C30B—H30D	109.3
H30A—C30A—H30B	107.9	H30C—C30B—H30D	108.0
			
C6A—C1A—C2A—C3A	−0.2 (3)	C6B—C1B—C2B—C3B	0.4 (3)
C7A—O1A—C3A—C2A	22.6 (2)	C7B—O1B—C3B—C2B	21.1 (2)
C7A—O1A—C3A—C4A	−159.49 (14)	C7B—O1B—C3B—C4B	−160.94 (14)
C1A—C2A—C3A—O1A	175.60 (16)	C1B—C2B—C3B—O1B	176.52 (15)
C1A—C2A—C3A—C4A	−2.2 (2)	C1B—C2B—C3B—C4B	−1.3 (2)
O1A—C3A—C4A—C5A	−175.61 (14)	O1B—C3B—C4B—C5B	−177.37 (14)
C2A—C3A—C4A—C5A	2.4 (2)	C2B—C3B—C4B—C5B	0.7 (2)
O1A—C3A—C4A—C13A	5.6 (2)	O1B—C3B—C4B—C13B	5.6 (2)
C2A—C3A—C4A—C13A	−176.36 (15)	C2B—C3B—C4B—C13B	−176.37 (15)
C10A—O2A—C5A—C6A	−2.7 (2)	C10B—O2B—C5B—C6B	−2.8 (2)
C10A—O2A—C5A—C4A	177.65 (14)	C10B—O2B—C5B—C4B	176.58 (13)
C3A—C4A—C5A—O2A	179.42 (14)	C3B—C4B—C5B—O2B	−178.51 (14)
C13A—C4A—C5A—O2A	−1.8 (2)	C13B—C4B—C5B—O2B	−1.5 (2)
C3A—C4A—C5A—C6A	−0.3 (2)	C3B—C4B—C5B—C6B	0.9 (2)
C13A—C4A—C5A—C6A	178.46 (15)	C13B—C4B—C5B—C6B	177.94 (15)
C2A—C1A—C6A—C5A	2.2 (3)	C2B—C1B—C6B—C5B	1.1 (3)
O2A—C5A—C6A—C1A	178.38 (16)	O2B—C5B—C6B—C1B	177.55 (15)
C4A—C5A—C6A—C1A	−2.0 (2)	C4B—C5B—C6B—C1B	−1.8 (2)
C3A—O1A—C7A—C9A	67.99 (18)	C3B—O1B—C7B—C8B	−169.65 (14)
C3A—O1A—C7A—C8A	−170.30 (14)	C3B—O1B—C7B—C9B	68.52 (19)
C5A—O2A—C10A—C11A	167.36 (14)	C5B—O2B—C10B—C11B	164.71 (14)
C5A—O2A—C10A—C12A	−72.79 (19)	C5B—O2B—C10B—C12B	−74.49 (18)
C5A—C4A—C13A—C14A	−96.12 (19)	C3B—C4B—C13B—C14B	73.2 (2)
C3A—C4A—C13A—C14A	82.6 (2)	C5B—C4B—C13B—C14B	−103.78 (18)
C5A—C4A—C13A—C18A	83.7 (2)	C3B—C4B—C13B—C18B	−105.83 (19)
C3A—C4A—C13A—C18A	−97.6 (2)	C5B—C4B—C13B—C18B	77.2 (2)
C18A—C13A—C14A—C15A	−1.1 (3)	C18B—C13B—C14B—C15B	−1.8 (2)
C4A—C13A—C14A—C15A	178.67 (16)	C4B—C13B—C14B—C15B	179.17 (15)
C13A—C14A—C15A—C16A	0.5 (3)	C13B—C14B—C15B—C16B	−0.3 (2)
C14A—C15A—C16A—C17A	0.1 (3)	C14B—C15B—C16B—C17B	1.2 (2)
C15A—C16A—C17A—C18A	0.0 (3)	C15B—C16B—C17B—C18B	0.0 (2)
C16A—C17A—C18A—C13A	−0.7 (2)	C14B—C13B—C18B—C17B	3.0 (2)
C16A—C17A—C18A—P1A	176.77 (13)	C4B—C13B—C18B—C17B	−178.03 (14)
C14A—C13A—C18A—C17A	1.2 (2)	C14B—C13B—C18B—P1B	−171.87 (12)
C4A—C13A—C18A—C17A	−178.58 (15)	C4B—C13B—C18B—P1B	7.1 (2)
C14A—C13A—C18A—P1A	−176.40 (12)	C16B—C17B—C18B—C13B	−2.1 (2)
C4A—C13A—C18A—P1A	3.8 (2)	C16B—C17B—C18B—P1B	172.39 (13)
C25A—P1A—C18A—C17A	29.19 (16)	C25B—P1B—C18B—C13B	−159.33 (13)
C19A—P1A—C18A—C17A	−79.14 (15)	C19B—P1B—C18B—C13B	93.19 (13)
C25A—P1A—C18A—C13A	−153.34 (13)	C25B—P1B—C18B—C17B	26.15 (16)
C19A—P1A—C18A—C13A	98.32 (13)	C19B—P1B—C18B—C17B	−81.32 (15)
C18A—P1A—C19A—C20A	69.43 (14)	C18B—P1B—C19B—C20B	72.74 (14)
C25A—P1A—C19A—C20A	−34.95 (15)	C25B—P1B—C19B—C20B	−31.67 (15)
C18A—P1A—C19A—C24A	−161.19 (13)	C18B—P1B—C19B—C24B	−158.96 (12)
C25A—P1A—C19A—C24A	94.43 (14)	C25B—P1B—C19B—C24B	96.63 (13)
C24A—C19A—C20A—C21A	54.5 (2)	C24B—C19B—C20B—C21B	54.6 (2)
P1A—C19A—C20A—C21A	−175.70 (13)	P1B—C19B—C20B—C21B	−176.42 (13)
C19A—C20A—C21A—C22A	−56.6 (2)	C19B—C20B—C21B—C22B	−55.9 (2)
C20A—C21A—C22A—C23A	57.2 (2)	C20B—C21B—C22B—C23B	56.4 (2)
C21A—C22A—C23A—C24A	−56.6 (2)	C21B—C22B—C23B—C24B	−56.9 (2)
C20A—C19A—C24A—C23A	−53.4 (2)	C22B—C23B—C24B—C19B	56.1 (2)
P1A—C19A—C24A—C23A	174.79 (13)	C20B—C19B—C24B—C23B	−54.35 (19)
C22A—C23A—C24A—C19A	54.8 (2)	P1B—C19B—C24B—C23B	174.40 (12)
C18A—P1A—C25A—C26A	64.78 (12)	C18B—P1B—C25B—C30B	−173.24 (12)
C19A—P1A—C25A—C26A	166.96 (11)	C19B—P1B—C25B—C30B	−72.42 (13)
C18A—P1A—C25A—C30A	−175.44 (12)	C18B—P1B—C25B—C26B	66.26 (13)
C19A—P1A—C25A—C30A	−73.26 (13)	C19B—P1B—C25B—C26B	167.08 (11)
C30A—C25A—C26A—C27A	56.37 (18)	C30B—C25B—C26B—C27B	55.71 (19)
P1A—C25A—C26A—C27A	177.09 (11)	P1B—C25B—C26B—C27B	177.10 (12)
C25A—C26A—C27A—C28A	−57.73 (19)	C25B—C26B—C27B—C28B	−56.7 (2)
C26A—C27A—C28A—C29A	56.0 (2)	C26B—C27B—C28B—C29B	56.3 (2)
C27A—C28A—C29A—C30A	−55.3 (2)	C27B—C28B—C29B—C30B	−56.1 (2)
C28A—C29A—C30A—C25A	55.2 (2)	C28B—C29B—C30B—C25B	56.1 (2)
C26A—C25A—C30A—C29A	−54.59 (19)	C26B—C25B—C30B—C29B	−55.11 (19)
P1A—C25A—C30A—C29A	−173.47 (12)	P1B—C25B—C30B—C29B	−174.39 (12)
