# Supplementary material for: Crystal structure of the RuPhos ligand
Source: Acta Crystallogr E Crystallogr Commun. 2021 Jan 26;77(Pt 2):171–4. doi: 10.1107/S2056989021000542 (PMC7869533; doi:10.1107/S2056989021000542)
Supplement: Supplementary file 3 [file e-77-00171-sup4.pdf]

Supporting Information

Crystal Structure of the RuPhos Ligand

Kurtis Carsch,* William Ho, Kai Hin Lui, Gregory Valtierra, Dilek K. Dogutan,* Daniel G. Nocera and Shao-Liang Zheng*

Department of Chemistry and Chemical Biology, Harvard University, 12 Oxford Street, Cambridge, MA 02138

* Corresponding author emails: kcarsch@g.harvard.edu, dkiper@fas.harvard.edu, and zheng@chemistry.harvard.edu

Table of Contents

A. Crystal and Models S3

Figure S1. Picture of mounted crystal S3

Figure S2. Ellipsoid model from CSD Mercury S4

Figure S3. Space-filling model S5

Figure S4. Reciprocal-lattice vectors along [100] S6

Figure S5. Reciprocal-lattice vectors along [010] S6

Figure S6. Reciprocal-lattice vectors along [010]. S7

B. Structural Metrics S4

Table S1. Fractional atomic coordinates and displacement parameters S8

Table S2. Atomic displacement parameters S12

Table S3. Bond lengths S14

Table S4. Bond angles S16

Table S5. Cone angle calculation results S22

Table S6. Distances between closest carbons on neighboring phenyls S22

A. Crystal and Models

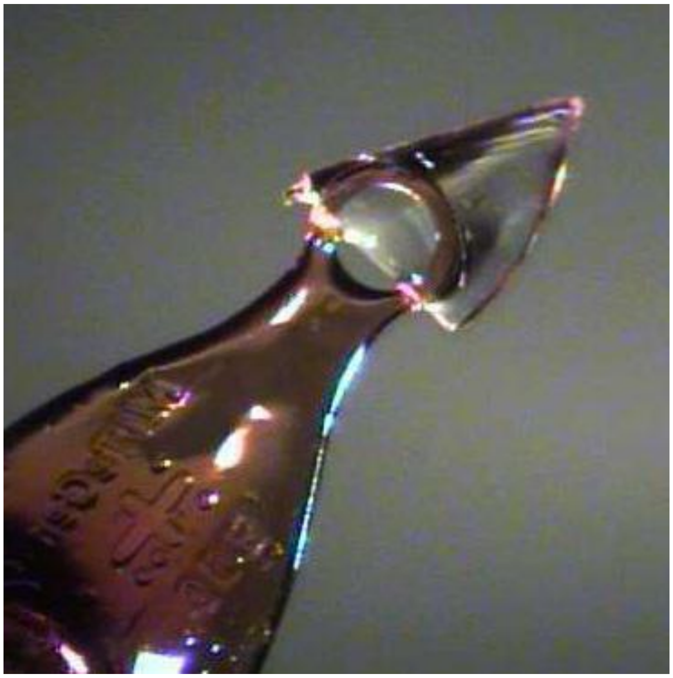

Figure S1. Mounted crystal of RuPhos.

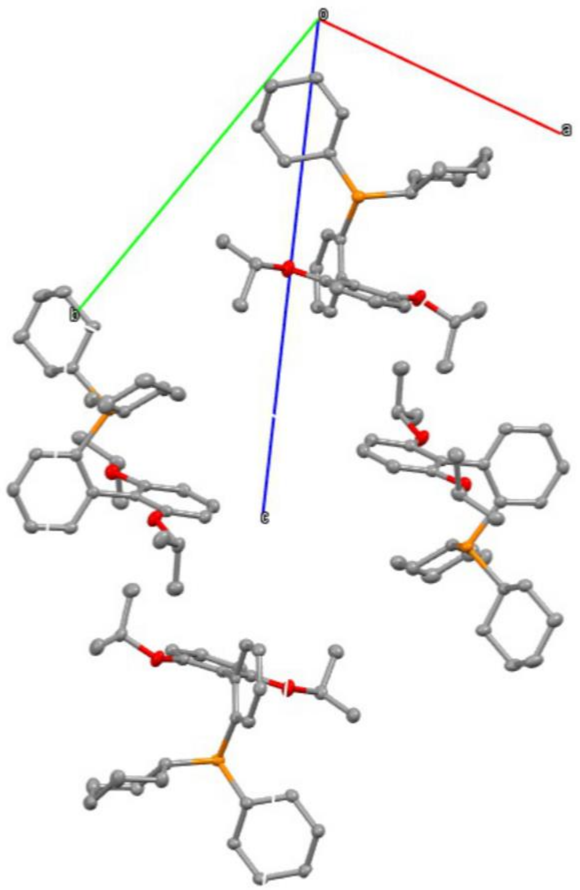

Figure S2. Ellipsoid model (CSD Mercury) of the crystal structure of RuPhos, demonstrating arrangement of biaryl substituents in a zigzag pattern.

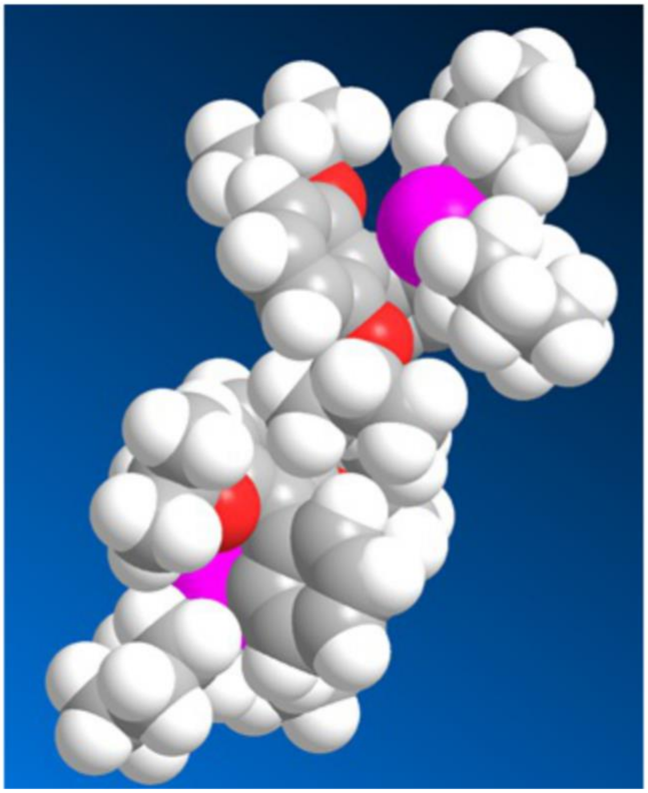

Figure S3. Space-filling model (CSD Mercury) of RuPhos asymmetric unit. Color scheme: P (pink), C (gray), H (white), O (red).

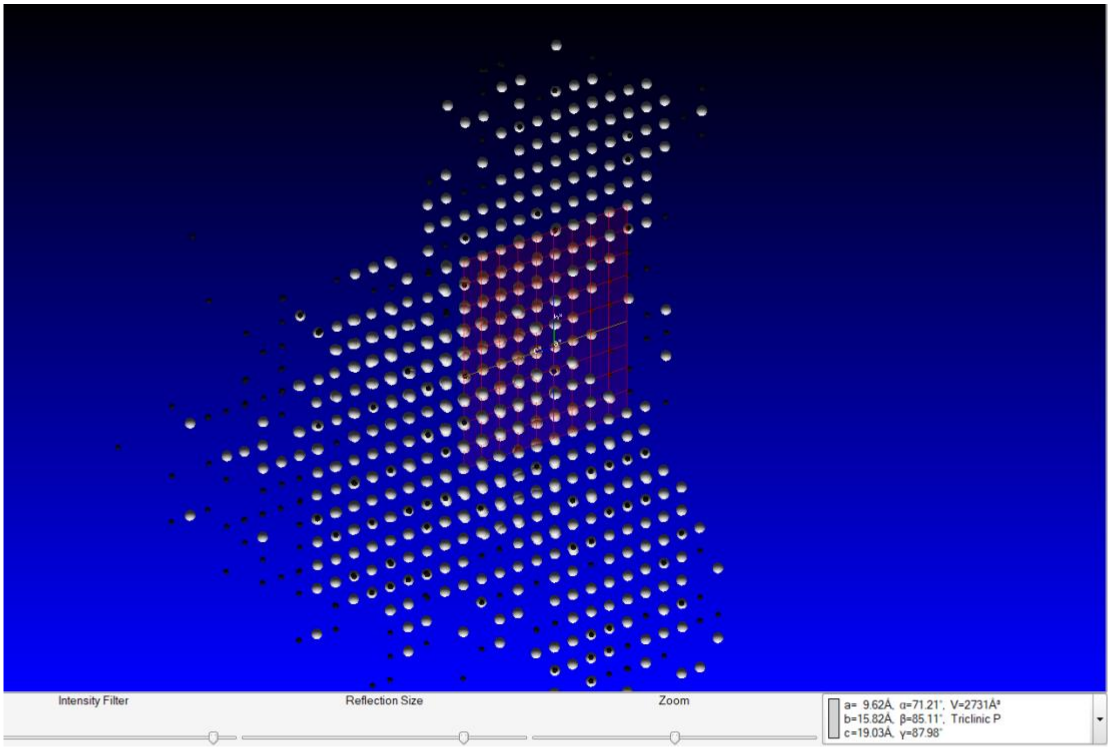

Figure S4. Reciprocal-lattice vectors along [100].

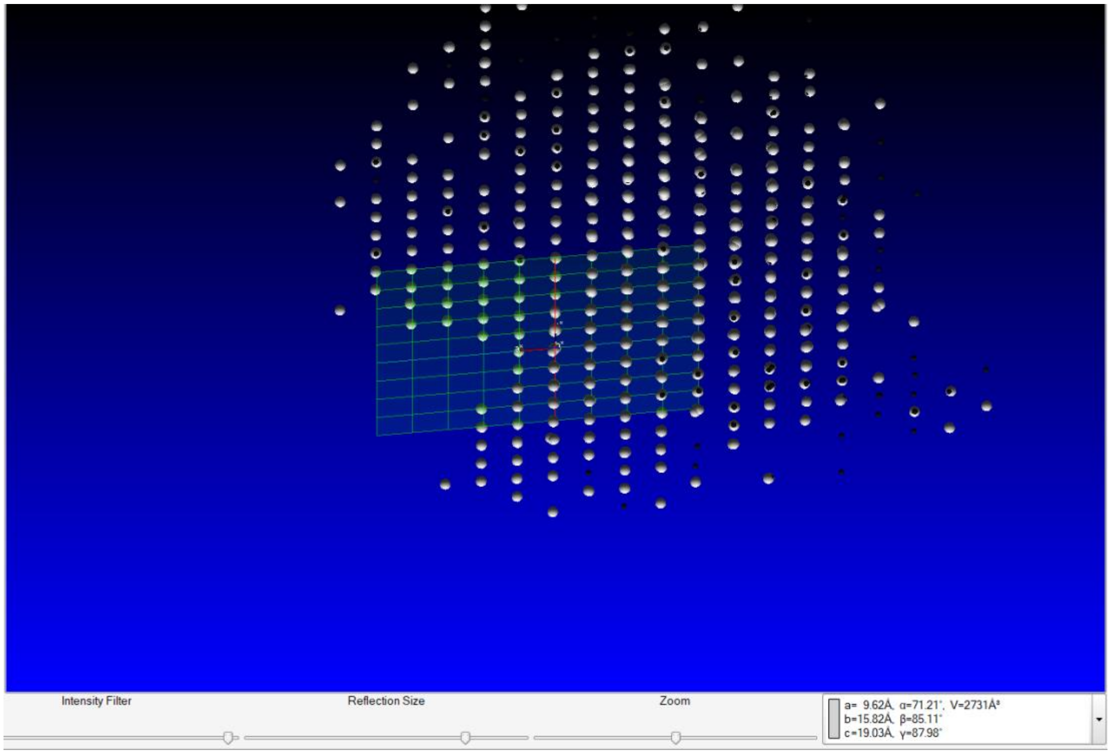

Figure S5. Reciprocal-lattice vectors along [010].

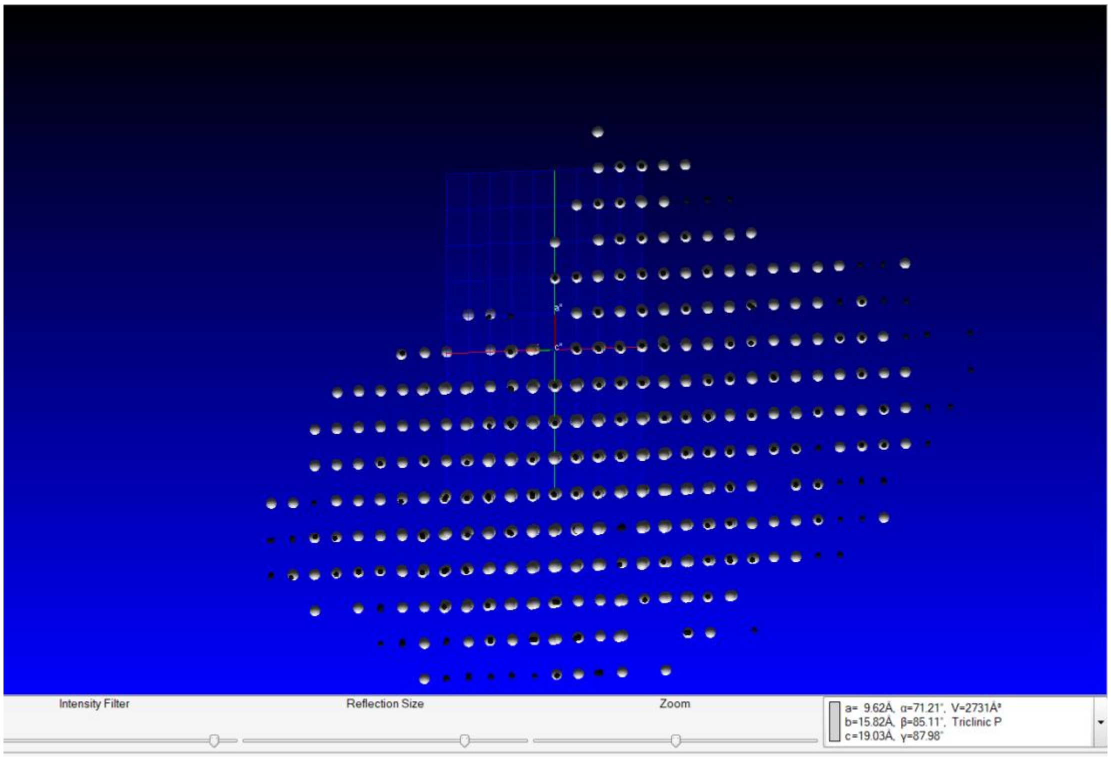

Figure S6. Reciprocal-lattice vectors along [010].

B. Structural Metrics

Table S1. Fractional atomic coordinates and isotropic or equivalent isotropic displacement parameters (Å^2) for RuPhos.

Atom	x	y	z	U_{iso}*/U_{eq}
P1A	0.44121(4)	0.24874(3)	0.10395(2)	0.02025(12)
O1A	0.73773(12)	0.26833(8)	0.22546(7)	0.0254(3)
O2A	0.38076(12)	0.46762(8)	0.12987(7)	0.0260(3)
C1A	0.76140(18)	0.49580(12)	0.09137(10)	0.0253(4)
H1A	0.828797	0.538226	0.062727	0.030*
C2A	0.80634(18)	0.41479(12)	0.13848(9)	0.0232(4)
H2A	0.903170	0.401717	0.142059	0.028*
C3A	0.70763(17)	0.35312(11)	0.18034(9)	0.0205(4)
C4A	0.56474(17)	0.37326(11)	0.17716(9)	0.0201(4)
C5A	0.52256(17)	0.45552(11)	0.12887(9)	0.0212 (4)
C6A	0.62045(18)	0.51673(12)	0.08484(9)	0.0238(4)
H6A	0.591592	0.571762	0.050958	0.029*
C7A	0.87460(17)	0.24929(12)	0.25305(9)	0.0226(4)
H7A	0.947689	0.268124	0.210533	0.027*
C8A	0.8784 (2)	0.14893(12)	0.28799(11)	0.0327(4)
H8AA	0.803622	0.130434	0.327973	0.049*
H8AB	0.968856	0.130520	0.308542	0.049*
H8AC	0.865337	0.120769	0.250117	0.049*
C9A	0.8942 (2)	0.29824(13)	0.30753(11)	0.0337(5)
H9AA	0.881824	0.362507	0.283121	0.051*
H9AB	0.988436	0.286470	0.324639	0.051*
H9AC	0.825244	0.277568	0.350252	0.051*
C10A	0.32140(18)	0.55063(11)	0.08509(10)	0.0234(4)
H10A	0.364353	0.567120	0.032781	0.028*
C11A	0.16772(18)	0.53064(12)	0.08720(10)	0.0286(4)
H11A	0.157521	0.479873	0.069211	0.043*
H11B	0.120351	0.583090	0.055317	0.043*
H11C	0.125984	0.516023	0.138452	0.043*
C12A	0.3421 (2)	0.62528(12)	0.11709(10)	0.0281(4)
H12A	0.302150	0.607829	0.168801	0.042*
H12B	0.295424	0.679661	0.087671	0.042*
H12C	0.442088	0.636519	0.115453	0.042*
C13A	0.45947(16)	0.30853(11)	0.22582(9)	0.0189(4)
C14A	0.42997(18)	0.30783(12)	0.29927(9)	0.0244(4)
H14A	0.478409	0.347259	0.316983	0.029*
C15A	0.33181(18)	0.25092(12)	0.34640(9)	0.0243(4)
H15A	0.312327	0.251473	0.396033	0.029*
C16A	0.26173(17)	0.19284(12)	0.32077(9)	0.0228(4)

Table S1 (cont'd). Fractional atomic coordinates and isotropic or equivalent isotropic displacement parameters (Å^2) for RuPhos.

Atom	x	y	z	U_{iso}*/U_{eq}
H16A	0.193853	0.153456	0.352835	0.027*
C17A	0.29094(17)	0.19236(11)	0.24816(9)	0.0219(4)
H17A	0.242364	0.152374	0.231117	0.026*
C18A	0.39021(16)	0.24941(11)	0.19962(9)	0.0191(4)
C19A	0.56208(17)	0.14958(11)	0.12748(9)	0.0225(4)
H19A	0.623416	0.158877	0.164187	0.027*
C20A	0.49358(18)	0.05907(12)	0.16617(11)	0.0277(4)
H20A	0.430588	0.046087	0.132477	0.033*
H20B	0.436622	0.061208	0.211426	0.033*
C21A	0.6032(2)	−0.01574(13)	0.18785(12)	0.0336(5)
H21A	0.660653	−0.005816	0.225178	0.040*
H21B	0.555675	−0.073817	0.210701	0.040*
C22A	0.6969(2)	−0.01855 (13)	0.12017(12)	0.0358(5)
H22A	0.768857	−0.065767	0.135702	0.043*
H22B	0.640685	−0.033274	0.084521	0.043*
C23A	0.7672(2)	0.07093(14)	0.08252(11)	0.0343(5)
H23A	0.824561	0.068569	0.037461	0.041*
H23B	0.830192	0.082695	0.116769	0.041*
C24A	0.66063(19)	0.14738(14)	0.06047(10)	0.0317(4)
H24A	0.710452	0.204885	0.039698	0.038*
H24B	0.605455	0.139840	0.021323	0.038*
C25A	0.27731(17)	0.20963(11)	0.07905(9)	0.0208(4)
H25A	0.247972	0.152025	0.117565	0.025*
C26A	0.16401(17)	0.28097(11)	0.07721(9)	0.0228(4)
H26A	0.147661	0.287411	0.127304	0.027*
H26B	0.197014	0.339067	0.042142	0.027*
C27A	0.02658(18)	0.25724(13)	0.05328(10)	0.0272(4)
H27A	−0.042876	0.305446	0.051484	0.033*
H27B	−0.010478	0.201445	0.090221	0.033*
C28A	0.04904(19)	0.24489(13)	−0.02343(10)	0.0287(4)
H28A	−0.039501	0.226291	−0.036863	0.034*
H28B	0.077098	0.302388	−0.061213	0.034*
C29A	0.16146(18)	0.17469(12)	−0.02343(10)	0.0265(4)
H29A	0.178402	0.170659	−0.074302	0.032*
H29B	0.128080	0.115811	0.010016	0.032*
C30A	0.29839(18)	0.19636(12)	0.00209(9)	0.0232(4)
H30A	0.337947	0.251408	−0.034815	0.028*
H30B	0.366119	0.147097	0.004464	0.028*
P1B	1.03926(4)	0.27391(3)	0.64698(2)	0.01980(12)
O1B	0.73186(12)	0.17442(8)	0.56160(6)	0.0247(3)

Table S1 (cont'd). Fractional atomic coordinates and isotropic or equivalent isotropic displacement parameters (Å^2) for RuPhos.

Atom	x	y	z	U_{iso}*/U_{eq}
O2B	1.08772(12)	0.37786(8)	0.45063(6)	0.0236(3)
C1B	0.70695(18)	0.41179 (12)	0.45028(9)	0.0243(4)
H1B	0.639525	0.457095	0.431652	0.029*
C2B	0.66229(18)	0.32608(12)	0.49049(9)	0.0232(4)
H2B	0.565622	0.312511	0.499134	0.028*
C3B	0.76187(17)	0.26046(11)	0.51789(9)	0.0198(4)
C4B	0.90492(17)	0.27906(11)	0.50426(9)	0.0185(4)
C5B	0.94642(17)	0.36631(11)	0.46270(9)	0.0201(4)
C6B	0.84761(18)	0.43300(12)	0.43657(9)	0.0238(4)
H6B	0.876068	0.492322	0.409687	0.029*
C7B	0.59642(17)	0.13792(12)	0.56147(10)	0.0239(4)
H7B	0.521795	0.179088	0.571536	0.029*
C8B	0.59304(19)	0.05138(13)	0.62519(11)	0.0335(5)
H8BA	0.667611	0.011687	0.615704	0.050*
H8BB	0.502508	0.022705	0.629810	0.050*
H8BC	0.606787	0.063489	0.671467	0.050*
C9B	0.5798 (2)	0.12544(13)	0.48696(11)	0.0323(4)
H9BA	0.589932	0.183135	0.447410	0.049*
H9BB	0.487089	0.101298	0.487438	0.049*
H9BC	0.651476	0.083819	0.477855	0.049*
C10B	1.14318(18)	0.46379(11)	0.40493(9)	0.0244(4)
H10B	1.093593	0.512342	0.420388	0.029*
C11B	1.29504(19)	0.46067(13)	0.42091(10)	0.0283(4)
H11D	1.301510	0.450503	0.474117	0.042*
H11E	1.339250	0.517506	0.391998	0.042*
H11F	1.342733	0.411976	0.406959	0.042*
C12B	1.1272(2)	0.47910(13)	0.32287(10)	0.0301(4)
H12D	1.178641	0.432585	0.307474	0.045*
H12E	1.164566	0.537774	0.293236	0.045*
H12F	1.028194	0.476796	0.315047	0.045*
C13B	1.01048(16)	0.20602(11)	0.53049(9)	0.0177(3)
C14B	1.03032(17)	0.14347(11)	0.49285(9)	0.0215(4)
H14B	0.976273	0.147910	0.452260	0.026*
C15B	1.12756(17)	0.07502(11)	0.51373(9)	0.0227(4)
H15B	1.139771	0.032658	0.487868	0.027*
C16B	1.20710(17)	0.06889(11)	0.57289(9)	0.0224(4)
H16B	1.275321	0.022894	0.587028	0.027*
C17B	1.18669(17)	0.13003(11)	0.61122(9)	0.0217(4)
H17B	1.241577	0.125313	0.651551	0.026*
C18B	1.08682(17)	0.19868(11)	0.59176(9)	0.0194(4)

Table S1 (cont'd). Fractional atomic coordinates and isotropic or equivalent isotropic displacement parameters (Å^2) for RuPhos.

Atom	x	y	z	U_{iso}*/U_{eq}
C19B	0.91541(17)	0.19627(11)	0.71832(9)	0.0223(4)
H19B	0.856780	0.169966	0.689800	0.027*
C20B	0.98146(18)	0.11693(12)	0.77526(10)	0.0286(4)
H20C	1.041014	0.082756	0.748730	0.034*
H20D	1.041788	0.139264	0.804967	0.034*
C21B	0.8713 (2)	0.05504(13)	0.82749(11)	0.0345(5)
H21C	0.817380	0.027502	0.798613	0.041*
H21D	0.918013	0.006570	0.865178	0.041*
C22B	0.7725(2)	0.10601(14)	0.86646(11)	0.0353(5)
H22C	0.699449	0.065258	0.897991	0.042*
H22D	0.824911	0.128745	0.899127	0.042*
C23B	0.70455(19)	0.18366(13)	0.80992(11)	0.0320(4)
H23C	0.643527	0.217229	0.836379	0.038*
H23D	0.645504	0.160359	0.780269	0.038*
C24B	0.81342(18)	0.24663(12)	0.75773(10)	0.0272(4)
H24C	0.866091	0.274836	0.786643	0.033*
H24D	0.765810	0.294559	0.720036	0.033*
C25B	1.20122(17)	0.27172(11)	0.69469(9)	0.0208(4)
H25B	1.228157	0.208342	0.720520	0.025*
C26B	1.31797(17)	0.31641(12)	0.63462(9)	0.0222(4)
H26C	1.334558	0.281686	0.599700	0.027*
H26D	1.287521	0.377284	0.605919	0.027*
C27B	1.45393(18)	0.32211(13)	0.66890(10)	0.0282(4)
H27C	1.524993	0.353401	0.629089	0.034*
H27D	1.489293	0.261125	0.693694	0.034*
C28B	1.4312 (2)	0.37213(14)	0.72561(10)	0.0321(4)
H28C	1.519260	0.372367	0.748948	0.039*
H28D	1.404605	0.434862	0.699914	0.039*
C29B	1.31674(19)	0.32821(13)	0.78577(10)	0.0289(4)
H29C	1.300406	0.363504	0.820269	0.035*
H29D	1.347692	0.267590	0.814789	0.035*
C30B	1.18020(18)	0.32175(12)	0.75184(9)	0.0245 (4)
H30C	1.144103	0.382614	0.727317	0.029*
H30D	1.109851	0.290248	0.791983	0.029*

Table S2. Atomic displacement parameters (Å^2) for RuPhos.

Atom	U_{11}	U_{22}	U_{33}	U_{12}	U_{13}	U_{23}
P1A	0.0213(2)	0.0221(2)	0.0174(2)	−0.00280(18)	−0.00069 (17)	−0.00635(18)
O1A	0.0190(6)	0.0217(7)	0.0317(7)	−0.0014(5)	−0.0075 (5)	−0.0017(5)
O2A	0.0212(6)	0.0212(7)	0.0308(7)	0.0014(5)	−0.0038 (5)	−0.0016(5)
C1A	0.0266(9)	0.0263(10)	0.0233(9)	−0.0079(8)	0.0009 (7)	−0.0082(8)
C2A	0.0209(9)	0.0256(10)	0.0242(9)	−0.0019(7)	−0.0023 (7)	−0.0092(8)
C3A	0.0228(9)	0.0212(9)	0.0187(8)	−0.0007(7)	−0.0051 (7)	−0.0070(7)
C4A	0.0230(9)	0.0201(9)	0.0188(8)	−0.0012(7)	−0.0026 (7)	−0.0081(7)
C5A	0.0212(9)	0.0226(9)	0.0214(9)	−0.0003(7)	−0.0021 (7)	−0.0092(7)
C6A	0.0287(10)	0.0217(9)	0.0206(9)	−0.0019(7)	−0.0018 (7)	−0.0058(7)
C7A	0.0172(8)	0.0266(10)	0.0229(9)	0.001(7)	−0.0047 (7)	−0.0059(8)
C8A	0.0289(10)	0.0289(11)	0.0387(11)	0.0028(8)	−0.0109 (8)	−0.0068(9)
C9A	0.0408(11)	0.0334(11)	0.0284(10)	0.003(9)	−0.0133 (9)	−0.0099(9)
C10A	0.0273(9)	0.0200(9)	0.0209(9)	0.0036(7)	−0.0048 (7)	−0.0035(7)
C11A	0.0271(10)	0.0286(10)	0.0315(10)	0.0048(8)	−0.0072 (8)	−0.0109(8)
C12A	0.0328(10)	0.0269(10)	0.0256(10)	0.0008(8)	−0.0022 (8)	−0.0099(8)
C13A	0.0183(8)	0.0171(9)	0.0202(8)	0.0036(7)	−0.0040 (7)	−0.0043(7)
C14A	0.0260(9)	0.0259(10)	0.0235(9)	−0.0003(7)	−0.0045 (7)	−0.0103(8)
C15A	0.0264(9)	0.0293(10)	0.0173(9)	0.0024(8)	−0.0030 (7)	−0.0076(8)
C16A	0.0206(9)	0.0240(9)	0.0200(9)	−0.0010(7)	0.0011 (7)	−0.0025(7)
C17A	0.0212(9)	0.0223(9)	0.0228(9)	−0.0019(7)	−0.0037 (7)	−0.0075(7)
C18A	0.0192(8)	0.0204(9)	0.0171(8)	0.0029(7)	−0.0031 (7)	−0.0049(7)
C19A	0.0211(9)	0.0258(10)	0.0217(9)	0.0004(7)	−0.0033 (7)	−0.0089(7)
C20A	0.0243(9)	0.0233(10)	0.0349(10)	−0.0005(7)	−0.0029 (8)	−0.0085(8)
C21A	0.0304(10)	0.0241(10)	0.0454(12)	0.0001(8)	−0.0067 (9)	−0.0091(9)
C22A	0.0331(11)	0.0382(12)	0.0455(12)	0.0123(9)	−0.0159 (9)	−0.0246(10)
C23A	0.0276(10)	0.0470(13)	0.0282(10)	0.0096(9)	−0.0017 (8)	−0.0130(9)
C24A	0.0287(10)	0.0406(12)	0.0240(10)	0.0065(9)	−0.0001 (8)	−0.0092(9)
C25A	0.0229(9)	0.0207(9)	0.0193(9)	−0.0012(7)	−0.0018 (7)	−0.0068(7)
C26A	0.0263(9)	0.0224(9)	0.0197(9)	0.0015(7)	−0.0021 (7)	−0.0070(7)
C27A	0.0252(9)	0.0332(11)	0.0260(10)	0.0055(8)	−0.0063 (7)	−0.0127(8)
C28A	0.0260(10)	0.0339(11)	0.0284(10)	0.0035(8)	−0.0081 (8)	−0.0122(8)
C29A	0.0299(10)	0.0300(10)	0.0231(9)	0.0006(8)	−0.0053 (7)	−0.0127(8)
C30A	0.0245(9)	0.0255(10)	0.0224(9)	0.0020(7)	−0.0047 (7)	−0.0110(8)
P1B	0.0204(2)	0.0215(2)	0.0189(2)	0.00027(18)	−0.00450 (17)	−0.00770(18)
O1B	0.0187(6)	0.0236(7)	0.0270(7)	−0.0037(5)	−0.0050 (5)	−0.0001(5)
O2B	0.0223(6)	0.0189(6)	0.0257(6)	−0.0036(5)	0.0004 (5)	−0.0021(5)
C1B	0.0262(9)	0.0231(10)	0.0243(9)	0.0051(7)	−0.0043 (7)	−0.0083(8)
C2B	0.0205(9)	0.0282(10)	0.0216(9)	0.0009(7)	−0.0032 (7)	−0.0086(8)
C3B	0.0234(9)	0.0197(9)	0.0166(8)	−0.0019(7)	−0.0022 (7)	−0.0057(7)
C4B	0.0216(9)	0.0205(9)	0.0152(8)	0.0001(7)	−0.0037 (6)	−0.0078(7)
C5B	0.0226(9)	0.0218(9)	0.0173(8)	−0.0015(7)	−0.0019 (7)	−0.0078(7)

Table S2 (cont'd). Atomic displacement parameters (Å2) for RuPhos.

Atom	U_{11}	U_{22}	U_{33}	U_{12}	U_{13}	U_{23}
C6B	0.0295(10)	0.0184(9)	0.0225(9)	−0.0001(7)	−0.0023 (7)	−0.0049(7)
C7B	0.0153(8)	0.0259(10)	0.0280(9)	−0.0026(7)	−0.0007 (7)	−0.0049(8)
C8B	0.0259(10)	0.0323(11)	0.0360(11)	−0.0073(8)	−0.0033 (8)	−0.0011(9)
C9B	0.0337(11)	0.0306(11)	0.0344(11)	−0.0029(8)	−0.0074 (8)	−0.0114(9)
C10B	0.0308(10)	0.0171(9)	0.0234(9)	−0.0063(7)	0.0022(7)	−0.0044(7)
C11B	0.0302(10)	0.0286(10)	0.0269(10)	−0.0099(8)	0.0027(8)	−0.0101(8)
C12B	0.0358(11)	0.0266(10)	0.0250(10)	−0.0033(8)	0.0004(8)	−0.0048(8)
C13B	0.0183(8)	0.0167(9)	0.0167(8)	−0.0039(7)	−0.0002(6)	−0.0032(7)
C14B	0.022(9)	0.0241(9)	0.0177(8)	−0.0049(7)	−0.0016(7)	−0.0059(7)
C15B	0.0250(9)	0.0202(9)	0.0236(9)	−0.0025(7)	0.0007(7)	−0.0086(7)
C16B	0.0212(9)	0.0184(9)	0.0255(9)	0.0003(7)	−0.0018(7)	−0.0041(7)
C17B	0.0198(9)	0.0239(9)	0.0208(9)	−0.0013(7)	−0.0058(7)	−0.0053(7)
C18B	0.0186(8)	0.0195(9)	0.0199(8)	−0.0033 (7)	0.0003(7)	−0.0061(7)
C19B	0.0202(9)	0.0247(9)	0.0242(9)	−0.0017(7)	−0.0044(7)	−0.0098(8)
C20B	0.0245(9)	0.0296(10)	0.0278(10)	−0.0016(8)	−0.0026(8)	−0.0036(8)
C21B	0.0325(11)	0.0329(11)	0.0313(11)	−0.0061(9)	−0.0015(8)	−0.0003(9)
C22B	0.0346(11)	0.0451(13)	0.0270(10)	−0.0155(9)	0.0035(8)	−0.0124(9)
C23B	0.0267(10)	0.0392(12)	0.0353(11)	−0.0069(8)	0.0036(8)	−0.0200(9)
C24B	0.0231(9)	0.0311(10)	0.0300(10)	−0.0011(8)	0.0000(8)	−0.0138(8)
C25B	0.0212(9)	0.0224(9)	0.0194(9)	−0.0008(7)	−0.0036(7)	−0.0069(7)
C26B	0.0241(9)	0.0236(9)	0.0191(9)	−0.0035(7)	−0.0017(7)	−0.0067(7)
C27B	0.0232(9)	0.0362(11)	0.0243(9)	−0.0073(8)	−0.0010(7)	−0.0081(8)
C28B	0.0306(10)	0.0409(12)	0.0262(10)	−0.0132(9)	−0.0050(8)	−0.0105(9)
C29B	0.0308(10)	0.0378(11)	0.0199(9)	−0.0059(8)	−0.0041(8)	−0.0108(8)
C30B	0.0251(9)	0.0303(10)	0.0201(9)	−0.0047(8)	−0.0026(7)	−0.0102(8)

Table S3. Bond lengths (Å) of RuPhos.

Bond	Length	Bond	Length
P1A—C18A	1.8482(16)	P1B—C18B	1.8482(17)
P1A—C25A	1.8645(17)	P1B—C25B	1.8624(17)
P1A—C19A	1.8762(17)	P1B—C19B	1.8771(17)
O1A—C3A	1.376(2)	O1B—C3B	1.373(2)
O1A—C7A	1.4444(19)	O1B—C7B	1.4437(19)
O2A—C5A	1.370(2)	O2B—C5B	1.367(2)
O2A—C10A	1.444(2)	O2B—C10B	1.449(2)
C1A—C2A	1.386(3)	C1B—C2B	1.388(2)
C1A—C6A	1.390(2)	C1B—C6B	1.388(2)
C1A—H1A	0.9500	C1B—H1B	0.9500
C2A—C3A	1.387(2)	C2B—C3B	1.390(2)
C2A—H2A	0.9500	C2B—H2B	0.9500
C3A—C4A	1.401(2)	C3B—C4B	1.402(2)
C4A—C5A	1.399(2)	C4B—C5B	1.404(2)
C4A—C13A	1.495(2)	C4B—C13B	1.499(2)
C5A—C6A	1.390(2)	C5B—C6B	1.391(2)
C6A—H6A	0.9500	C6B—H6B	0.9500
C7A—C9A	1.507(2)	C7B—C8B	1.508(2)
C7A—C8A	1.511(2)	C7B—C9B	1.516(3)
C7A—H7A	1.0000	C7B—H7B	1.0000
C8A—H8AA	0.9800	C8B—H8BA	0.9800
C8A—H8AB	0.9800	C8B—H8BB	0.9800
C8A—H8AC	0.9800	C8B—H8BC	0.9800
C9A—H9AA	0.9800	C9B—H9BA	0.9800
C9A—H9AB	0.9800	C9B—H9BB	0.9800
C9A—H9AC	0.9800	C9B—H9BC	0.9800
C10A—C11A	1.517(2)	C10B—C11B	1.513(2)
C10A—C12A	1.518(2)	C10B—C12B	1.522(2)
C10A—H10A	1.0000	C10B—H10B	1.0000
C11A—H11A	0.9800	C11B—H11D	0.9800
C11A—H11B	0.9800	C11B—H11E	0.9800
C11A—H11C	0.9800	C11B—H11F	0.9800
C12A—H12A	0.9800	C12B—H12D	0.9800
C12A—H12B	0.9800	C12B—H12E	0.9800
C12A—H12C	0.9800	C12B—H12F	0.9800
C13A—C14A	1.399(2)	C13B—C14B	1.396(2)
C13A—C18A	1.403(2)	C13B—C18B	1.402(2)
C14A—C15A	1.379(2)	C14B—C15B	1.385(2)
C14A—H14A	0.9500	C14B—H14B	0.9500
C15A—C16A	1.387(2)	C15B—C16B	1.390(2)
C15A—H15A	0.9500	C15B—H15B	0.9500

Table S3 (cont'd). Bond lengths (Å) of RuPhos.

Bond	Length	Bond	Length
C16A—C17A	1.389(2)	C16B—C17B	1.387(2)
C16A—H16A	0.9500	C16B—H16B	0.9500
C17A—C18A	1.397(2)	C17B—C18B	1.402(2)
C17A—H17A	0.9500	C17B—H17B	0.9500
C19A—C20A	1.527(2)	C19B—C20B	1.529(2)
C19A—C24A	1.532(2)	C19B—C24B	1.538(2)
C19A—H19A	1.0000	C19B—H19B	1.0000
C20A—C21A	1.534(3)	C20B—C21B	1.528(2)
C20A—H20A	0.9900	C20B—H20C	0.9900
C20A—H20B	0.9900	C20B—H20D	0.9900
C21A—C22A	1.520(3)	C21B—C22B	1.521(3)
C21A—H21A	0.9900	C21B—H21C	0.9900
C21A—H21B	0.9900	C21B—H21D	0.9900
C22A—C23A	1.518(3)	C22B—C23B	1.518(3)
C22A—H22A	0.9900	C22B—H22C	0.9900
C22A—H22B	0.9900	C22B—H22D	0.9900
C23A—C24A	1.532(3)	C23B—C24B	1.528(2)
C23A—H23A	0.9900	C23B—H23C	0.9900
C23A—H23B	0.9900	C23B—H23D	0.9900
C24A—H24A	0.9900	C24B—H24C	0.9900
C24A—H24B	0.9900	C24B—H24D	0.9900
C25A—C26A	1.535(2)	C25B—C30B	1.535(2)
C25A—C30A	1.541(2)	C25B—C26B	1.544(2)
C25A—H25A	1.0000	C25B—H25B	1.0000
C26A—C27A	1.531(2)	C26B—C27B	1.528(2)
C26A—H26A	0.9900	C26B—H26C	0.9900
C26A—H26B	0.9900	C26B—H26D	0.9900
C27A—C28A	1.531(2)	C27B—C28B	1.529(3)
C27A—H27A	0.9900	C27B—H27C	0.9900
C27A—H27B	0.9900	C27B—H27D	0.9900
C28A—C29A	1.522(2)	C28B—C29B	1.527(2)
C28A—H28A	0.9900	C28B—H28C	0.9900
C28A—H28B	0.9900	C28B—H28D	0.9900
C29A—C30A	1.528(2)	C29B—C30B	1.532(2)
C29A—H29A	0.9900	C29B—H29C	0.9900
C29A—H29B	0.9900	C29B—H29D	0.9900
C30A—H30A	0.9900	C30B—H30C	0.9900
C30A—H30B	0.9900	C30B—H30D	0.9900

Table S4. Bond angles (°) of RuPhos.

Bond	Angle	Bond	Angle
C18A—P1A—C25A	101.31(7)	C18B—P1B—C25B	101.86(8)
C18A—P1A—C19A	98.31(7)	C18B—P1B—C19B	97.03(7)
C25A—P1A—C19A	106.07(8)	C25B—P1B—C19B	105.46 (8)
C3A—O1A—C7A	119.19(12)	C3B—O1B—C7B	119.71(13)
C5A—O2A—C10A	120.37(13)	C5B—O2B—C10B	119.60(13)
C2A—C1A—C6A	121.85(16)	C2B—C1B—C6B	121.67(16)
C2A—C1A—H1A	119.1	C2B—C1B—H1B	119.2
C6A—C1A—H1A	119.1	C6B—C1B—H1B	119.2
C1A—C2A—C3A	118.90(16)	C1B—C2B—C3B	118.66(16)
C1A—C2A—H2A	120.6	C1B—C2B—H2B	120.7
C3A—C2A—H2A	120.6	C3B—C2B—H2B	120.7
O1A—C3A—C2A	124.80(15)	O1B—C3B—C2B	124.58(15)
O1A—C3A—C4A	114.40(14)	O1B—C3B—C4B	114.10(14)
C2A—C3A—C4A	120.76(15)	C2B—C3B—C4B	121.29(15)
C5A—C4A—C3A	118.97(15)	C3B—C4B—C5B	118.52(15)
C5A—C4A—C13A	120.65(15)	C3B—C4B—C13B	120.34(14)
C3A—C4A—C13A	120.36(15)	C5B—C4B—C13B	121.08(14)
O2A—C5A—C6A	125.33(15)	O2B—C5B—C6B	124.82(15)
O2A—C5A—C4A	113.90(14)	O2B—C5B—C4B	114.52(14)
C6A—C5A—C4A	120.76(16)	C6B—C5B—C4B	120.66(15)
C1A—C6A—C5A	118.68(16)	C1B—C6B—C5B	119.18(16)
C1A—C6A—H6A	120.7	C1B—C6B—H6B	120.4
C5A—C6A—H6A	120.7	C5B—C6B—H6B	120.4
O1A—C7A—C9A	110.12(14)	O1B—C7B—C8B	104.22(14)
O1A—C7A—C8A	104.46(13)	O1B—C7B—C9B	110.32(14)
C9A—C7A—C8A	113.08(15)	C8B—C7B—C9B	113.21(16)
O1A—C7A—H7A	109.7	O1B—C7B—H7B	109.6
C9A—C7A—H7A	109.7	C8B—C7B—H7B	109.6
C8A—C7A—H7A	109.7	C9B—C7B—H7B	109.6
C7A—C8A—H8AA	109.5	C7B—C8B—H8BA	109.5
C7A—C8A—H8AB	109.5	C7B—C8B—H8BB	109.5
H8AA—C8A—H8AB	109.5	H8BA—C8B—H8BB	109.5
C7A—C8A—H8AC	109.5	C7B—C8B—H8BC	109.5
H8AA—C8A—H8AC	109.5	H8BA—C8B—H8BC	109.5
H8AB—C8A—H8AC	109.5	H8BB—C8B—H8BC	109.5
C7A—C9A—H9AA	109.5	C7B—C9B—H9BA	109.5
C7A—C9A—H9AB	109.5	C7B—C9B—H9BB	109.5
H9AA—C9A—H9AB	109.5	H9BA—C9B—H9BB	109.5
C7A—C9A—H9AC	109.5	C7B—C9B—H9BC	109.5
H9AA—C9A—H9AC	109.5	H9BA—C9B—H9BC	109.5
H9AB—C9A—H9AC	109.5	H9BB—C9B—H9BC	109.5

Table S4 (cont'd). Bond angles (°) of RuPhos.

Bond	Angle	Bond	Angle
O2A—C10A—C11A	104.29(13)	O2B—C10B—C11B	105.04(14)
O2A—C10A—C12A	111.20(14)	O2B—C10B—C12B	111.04(14)
C11A—C10A—C12A	111.12(15)	C11B—C10B—C12B	111.61(15)
O2A—C10A—H10A	110.0	O2B—C10B—H10B	109.7
C11A—C10A—H10A	110.0	C11B—C10B—H10B	109.7
C12A—C10A—H10A	110.0	C12B—C10B—H10B	109.7
C10A—C11A—H11A	109.5	C10B—C11B—H11D	109.5
C10A—C11A—H11B	109.5	C10B—C11B—H11E	109.5
H11A—C11A—H11B	109.5	H11D—C11B—H11E	109.5
C10A—C11A—H11C	109.5	C10B—C11B—H11F	109.5
H11A—C11A—H11C	109.5	H11D—C11B—H11F	109.5
H11B—C11A—H11C	109.5	H11E—C11B—H11F	109.5
C10A—C12A—H12A	109.5	C10B—C12B—H12D	109.5
C10A—C12A—H12B	109.5	C10B—C12B—H12E	109.5
H12A—C12A—H12B	109.5	H12D—C12B—H12E	109.5
C10A—C12A—H12C	109.5	C10B—C12B—H12F	109.5
H12A—C12A—H12C	109.5	H12D—C12B—H12F	109.5
H12B—C12A—H12C	109.5	H12E—C12B—H12F	109.5
C14A—C13A—C18A	119.78(15)	C14B—C13B—C18B	119.97(15)
C14A—C13A—C4A	118.46(15)	C14B—C13B—C4B	118.23(14)
C18A—C13A—C4A	121.76(14)	C18B—C13B—C4B	121.79(14)
C15A—C14A—C13A	121.16(16)	C15B—C14B—C13B	121.06(16)
C15A—C14A—H14A	119.4	C15B—C14B—H14B	119.5
C13A—C14A—H14A	119.4	C13B—C14B—H14B	119.5
C14A—C15A—C16A	119.48(16)	C14B—C15B—C16B	119.40(16)
C14A—C15A—H15A	120.3	C14B—C15B—H15B	120.3
C16A—C15A—H15A	120.3	C16B—C15B—H15B	120.3
C15A—C16A—C17A	119.91(15)	C17B—C16B—C15B	119.89(16)
C15A—C16A—H16A	120.0	C17B—C16B—H16B	120.1
C17A—C16A—H16A	120.0	C15B—C16B—H16B	120.1
C16A—C17A—C18A	121.45(16)	C16B—C17B—C18B	121.45(16)
C16A—C17A—H17A	119.3	C16B—C17B—H17B	119.3
C18A—C17A—H17A	119.3	C18B—C17B—H17B	119.3
C17A—C18A—C13A	118.21(15)	C13B—C18B—C17B	118.16(15)
C17A—C18A—P1A	123.97(13)	C13B—C18B—P1B	117.90(12)
C13A—C18A—P1A	117.78(12)	C17B—C18B—P1B	123.72(13)
C20A—C19A—C24A	111.30(15)	C20B—C19B—C24B	110.52(15)
C20A—C19A—P1A	116.03(12)	C20B—C19B—P1B	116.21(12)
C24A—C19A—P1A	112.09(12)	C24B—C19B—P1B	111.95(12)
C20A—C19A—H19A	105.5	C20B—C19B—H19B	105.8
C24A—C19A—H19A	105.5	C24B—C19B—H19B	105.8

Table S4 (cont'd). Bond angles (°) of RuPhos.

Bond	Angle	Bond	Angle
P1A—C19A—H19A	105.5	P1B—C19B—H19B	105.8
C19A—C20A—C21A	111.30(14)	C21B—C20B—C19B	111.89(15)
C19A—C20A—H20A	109.4	C21B—C20B—H20C	109.2
C21A—C20A—H20A	109.4	C19B—C20B—H20C	109.2
C19A—C20A—H20B	109.4	C21B—C20B—H20D	109.2
C21A—C20A—H20B	109.4	C19B—C20B—H20D	109.2
H20A—C20A—H20B	108.0	H20C—C20B—H20D	107.9
C22A—C21A—C20A	110.94(16)	C22B—C21B—C20B	111.02(16)
C22A—C21A—H21A	109.5	C22B—C21B—H21C	109.4
C20A—C21A—H21A	109.5	C20B—C21B—H21C	109.4
C22A—C21A—H21B	109.5	C22B—C21B—H21D	109.4
C20A—C21A—H21B	109.5	C20B—C21B—H21D	109.4
H21A—C21A—H21B	108.0	H21C—C21B—H21D	108.0
C23A—C22A—C21A	110.46(16)	C23B—C22B—C21B	110.62(16)
C23A—C22A—H22A	109.6	C23B—C22B—H22C	109.5
C21A—C22A—H22A	109.6	C21B—C22B—H22C	109.5
C23A—C22A—H22B	109.6	C23B—C22B—H22D	109.5
C21A—C22A—H22B	109.6	C21B—C22B—H22D	109.5
H22A—C22A—H22B	108.1	H22C—C22B—H22D	108.1
C22A—C23A—C24A	111.86(16)	C22B—C23B—C24B	111.55(15)
C22A—C23A—H23A	109.2	C22B—C23B—H23C	109.3
C24A—C23A—H23A	109.2	C24B—C23B—H23C	109.3
C22A—C23A—H23B	109.2	C22B—C23B—H23D	109.3
C24A—C23A—H23B	109.2	C24B—C23B—H23D	109.3
H23A—C23A—H23B	107.9	H23C—C23B—H23D	108.0
C19A—C24A—C23A	111.26(15)	C23B—C24B—C19B	111.15(15)
C19A—C24A—H24A	109.4	C23B—C24B—H24C	109.4
C23A—C24A—H24A	109.4	C19B—C24B—H24C	109.4
C19A—C24A—H24B	109.4	C23B—C24B—H24D	109.4
C23A—C24A—H24B	109.4	C19B—C24B—H24D	109.4
H24A—C24A—H24B	108.0	H24C—C24B—H24D	108.0
C26A—C25A—C30A	109.39(14)	C30B—C25B—C26B	109.85(14)
C26A—C25A—P1A	107.90(11)	C30B—C25B—P1B	111.27(11)
C30A—C25A—P1A	110.88(11)	C26B—C25B—P1B	107.78(11)
C26A—C25A—H25A	109.5	C30B—C25B—H25B	109.3
C30A—C25A—H25A	109.5	C26B—C25B—H25B	109.3
P1A—C25A—H25A	109.5	P1B—C25B—H25B	109.3
C27A—C26A—C25A	111.96(14)	C27B—C26B—C25B	111.70(14)
C27A—C26A—H26A	109.2	C27B—C26B—H26C	109.3
C25A—C26A—H26A	109.2	C25B—C26B—H26C	109.3
C27A—C26A—H26B	109.2	C27B—C26B—H26D	109.3

Table S4 (cont'd). Bond angles (°) of RuPhos.

Bond	Angle	Bond	Angle
C25A—C26A—H26B	109.2	C25B—C26B—H26D	109.3
H26A—C26A—H26B	107.9	H26C—C26B—H26D	107.9
C26A—C27A—C28A	110.49(14)	C26B—C27B—C28B	110.84(15)
C26A—C27A—H27A	109.6	C26B—C27B—H27C	109.5
C28A—C27A—H27A	109.6	C28B—C27B—H27C	109.5
C26A—C27A—H27B	109.6	C26B—C27B—H27D	109.5
C28A—C27A—H27B	109.6	C28B—C27B—H27D	109.5
H27A—C27A—H27B	108.1	H27C—C27B—H27D	108.1
C29A—C28A—C27A	110.67(15)	C29B—C28B—C27B	110.81(15)
C29A—C28A—H28A	109.5	C29B—C28B—H28C	109.5
C27A—C28A—H28A	109.5	C27B—C28B—H28C	109.5
C29A—C28A—H28B	109.5	C29B—C28B—H28D	109.5
C27A—C28A—H28B	109.5	C27B—C28B—H28D	109.5
H28A—C28A—H28B	108.1	H28C—C28B—H28D	108.1
C28A—C29A—C30A	112.09(15)	C28B—C29B—C30B	111.36(15)
C28A—C29A—H29A	109.2	C28B—C29B—H29C	109.4
C30A—C29A—H29A	109.2	C30B—C29B—H29C	109.4
C28A—C29A—H29B	109.2	C28B—C29B—H29D	109.4
C30A—C29A—H29B	109.2	C30B—C29B—H29D	109.4
H29A—C29A—H29B	107.9	H29C—C29B—H29D	108.0
C29A—C30A—C25A	111.79 (14)	C29B—C30B—C25B	111.57 (14)
C29A—C30A—H30A	109.3	C29B—C30B—H30C	109.3
C25A—C30A—H30A	109.3	C25B—C30B—H30C	109.3
C29A—C30A—H30B	109.3	C29B—C30B—H30D	109.3
C25A—C30A—H30B	109.3	C25B—C30B—H30D	109.3
H30A—C30A—H30B	107.9	H30C—C30B—H30D	108.0
C6A—C1A—C2A—C3A	−0.2(3)	C6B—C1B—C2B—C3B	0.4(3)
C7A—O1A—C3A—C2A	22.6(2)	C7B—O1B—C3B—C2B	21.1(2)
C7A—O1A—C3A—C4A	−159.49(14)	C7B—O1B—C3B—C4B	−160.94(14)
C1A—C2A—C3A—O1A	175.60(16)	C1B—C2B—C3B—O1B	176.52(15)
C1A—C2A—C3A—C4A	−2.2(2)	C1B—C2B—C3B—C4B	−1.3(2)
O1A—C3A—C4A—C5A	−175.61(14)	O1B—C3B—C4B—C5B	−177.37(14)
C2A—C3A—C4A—C5A	2.4(2)	C2B—C3B—C4B—C5B	0.7 (2)
O1A—C3A—C4A—C13A	5.6(2)	O1B—C3B—C4B—C13B	5.6 (2)
C2A—C3A—C4A—C13A	−176.36(15)	C2B—C3B—C4B—C13B	−176.37 (15)
C10A—O2A—C5A—C6A	−2.7(2)	C10B—O2B—C5B—C6B	−2.8(2)
C10A—O2A—C5A—C4A	177.65(14)	C10B—O2B—C5B—C4B	176.58(13)
C3A—C4A—C5A—O2A	179.42(14)	C3B—C4B—C5B—O2B	−178.51(14)
C13A—C4A—C5A—O2A	−1.8(2)	C13B—C4B—C5B—O2B	−1.5(2)
C3A—C4A—C5A—C6A	−0.3(2)	C3B—C4B—C5B—C6B	0.9(2)
C13A—C4A—C5A—C6A	178.46(15)	C13B—C4B—C5B—C6B	177.94(15)

Table S4 (cont'd). Bond angles (°) of RuPhos.

Bond	Angle	Bond	Angle
C2A—C1A—C6A—C5A	2.2(3)	C2B—C1B—C6B—C5B	1.1 (3)
O2A—C5A—C6A—C1A	178.38(16)	O2B—C5B—C6B—C1B	177.55(15)
C4A—C5A—C6A—C1A	−2.0(2)	C4B—C5B—C6B—C1B	−1.8 (2)
C3A—O1A—C7A—C9A	67.99(18)	C3B—O1B—C7B—C8B	−169.65 (14)
C3A—O1A—C7A—C8A	−170.30(14)	C3B—O1B—C7B—C9B	68.52(19)
C5A—O2A—C10A—C11A	167.36(14)	C5B—O2B—C10B—C11B	164.71(14)
C5A—O2A—C10A—C12A	−72.79(19)	C5B—O2B—C10B—C12B	−74.49(18)
C5A—C4A—C13A—C14A	−96.12(19)	C3B—C4B—C13B—C14B	73.2(2)
C3A—C4A—C13A—C14A	82.6(2)	C5B—C4B—C13B—C14B	−103.78(18)
C5A—C4A—C13A—C18A	83.7(2)	C3B—C4B—C13B—C18B	−105.83(19)
C3A—C4A—C13A—C18A	−97.6(2)	C5B—C4B—C13B—C18B	77.2(2)
C18A—C13A—C14A—C15A	−1.1(3)	C18B—C13B—C14B—C15B	−1.8(2)
C4A—C13A—C14A—C15A	178.67(16)	C4B—C13B—C14B—C15B	179.17(15)
C13A—C14A—C15A—C16A	0.5(3)	C13B—C14B—C15B—C16B	−0.3(2)
C14A—C15A—C16A—C17A	0.1(3)	C14B—C15B—C16B—C17B	1.2(2)
C15A—C16A—C17A—C18A	0.0(3)	C15B—C16B—C17B—C18B	0.0(2)
C16A—C17A—C18A—C13A	−0.7(2)	C14B—C13B—C18B—C17B	3.0(2)
C16A—C17A—C18A—P1A	176.77(13)	C4B—C13B—C18B—C17B	−178.03(14)
C14A—C13A—C18A—C17A	1.2(2)	C14B—C13B—C18B—P1B	−171.87(12)
C4A—C13A—C18A—C17A	−178.58(15)	C4B—C13B—C18B—P1B	7.1(2)
C14A—C13A—C18A—P1A	−176.40(12)	C16B—C17B—C18B—C13B	−2.1(2)
C4A—C13A—C18A—P1A	3.8(2)	C16B—C17B—C18B—P1B	172.39(13)
C25A—P1A—C18A—C17A	29.19(16)	C25B—P1B—C18B—C13B	−159.33(13)
C19A—P1A—C18A—C17A	−79.14(15)	C19B—P1B—C18B—C13B	93.19(13)
C25A—P1A—C18A—C13A	−153.34(13)	C25B—P1B—C18B—C17B	26.15(16)
C19A—P1A—C18A—C13A	98.32(14)	C19B—P1B—C18B—C17B	−81.32(15)
C18A—P1A—C19A—C20A	69.43(14)	C18B—P1B—C19B—C20B	72.74(14)
C25A—P1A—C19A—C20A	−34.95(15)	C25B—P1B—C19B—C20B	−31.67(15)
C18A—P1A—C19A—C24A	−161.19(13)	C18B—P1B—C19B—C24B	−158.96(12)
C25A—P1A—C19A—C24A	94.43(14)	C25B—P1B—C19B—C24B	96.63(13)
C24A—C19A—C20A—C21A	54.5(2)	C24B—C19B—C20B—C21B	54.6(2)
P1A—C19A—C20A—C21A	−175.70(13)	P1B—C19B—C20B—C21B	−176.42(13)
C19A—C20A—C21A—C22A	−56.6(2)	C19B—C20B—C21B—C22B	−55.9(2)
C20A—C21A—C22A—C23A	57.2(2)	C20B—C21B—C22B—C23B	56.4(2)
C21A—C22A—C23A—C24A	−56.6(2)	C21B—C22B—C23B—C24B	−56.9(2)
C20A—C19A—C24A—C23A	−53.4(2)	C22B—C23B—C24B—C19B	56.1(2)
P1A—C19A—C24A—C23A	174.79(13)	C20B—C19B—C24B—C23B	−54.35(19)
C22A—C23A—C24A—C19A	54.8(2)	P1B—C19B—C24B—C23B	174.40(12)
C18A—P1A—C25A—C26A	64.78(12)	C18B—P1B—C25B—C30B	−173.24(12)
C19A—P1A—C25A—C26A	166.96(11)	C19B—P1B—C25B—C30B	−72.42(13)
C18A—P1A—C25A—C30A	−175.44(12)	C18B—P1B—C25B—C26B	66.26(13)

Table S4 (cont'd). Bond angles (°) of RuPhos.

Bond	Angle	Bond	Angle
C19A—P1A—C25A—C30A	−73.26(13)	C19B—P1B—C25B—C26B	167.08(11)
C30A—C25A—C26A—C27A	56.37(18)	C30B—C25B—C26B—C27B	55.71(19)
P1A—C25A—C26A—C27A	177.09(11)	P1B—C25B—C26B—C27B	177.10(12)
C25A—C26A—C27A—C28A	−57.73(19)	C25B—C26B—C27B—C28B	−56.7(2)
C26A—C27A—C28A—C29A	56.0(2)	C26B—C27B—C28B—C29B	56.3(2)
C27A—C28A—C29A—C30A	−55.3(2)	C27B—C28B—C29B—C30B	−56.1(2)
C28A—C29A—C30A—C25A	55.2(2)	C28B—C29B—C30B—C25B	56.1(2)
C26A—C25A—C30A—C29A	−54.59(19)	C26B—C25B—C30B—C29B	−55.11(19)
P1A—C25A—C30A—C29A	−173.47(12)	P1B—C25B—C30B—C29B	−174.39(12)

Table S5. Cone angle (°) calculations for Pd-RuPhos, free RuPhos, and free BrettPhos.

Substituent	Angle	Distance	Substituent Half Cone Angle	Overall Cone Angle
Pd-RuPhos	135.167	4.634(20)	147.63(5)	198.06(5)
Cy1	64.608	5.369(20)	74.14(4)	
Cy2	63.423	5.378(20)	75.32(4)	
free RuPhos	141.032	3.898(20)	155.89(8)	201.53(6)
Cy1	58.555	5.542(20)	68.95(4)	
Cy2	66.674	5.343(20)	77.46(4)	
free BrettPhos	154.84	5.921(20)	164.55(3)	220.28(12)
Cy1	64.27	5.291(20)	75.16(4)	
Cy2	69.04	2.70 (20)	90.7(2)	

Table S6. Distances (Å) between closest carbons on neighboring RuPhos phenyls.

C—C Atoms	Distance
C23A·····C27A	3.801(3)
C23B·····C27B	3.841(3)
C23A·····C27A	3.801(3)
C23B·····C27B	3.841(3)
C21B·····C20A	3.977(3)
C22A·····C20B	3.829(3)
C22B·····C20A	3.942(3)
C23B·····C27B	3.841(3)
